# Supplementary material for: Extensive cone-dependent spectral opponency within a discrete zone of the lateral geniculate nucleus supporting mouse color vision
Source: Curr Biol. 2021 Aug 9;31(15):3391–3400.e4. doi: 10.1016/j.cub.2021.05.024 (PMC8360768; doi:10.1016/j.cub.2021.05.024)
Supplement: Document S1. Figures S1–S4 [file mmc1.pdf]

Current Biology, Volume *31*

Supplemental Information

Extensive cone-dependent spectral opponency within a discrete zone of the lateral geniculate nucleus supporting mouse color vision

Josh W. Mouland, Abigail Pienaar, Christopher Williams, Alex J. Watson, Robert J. Lucas, and Timothy M. Brown

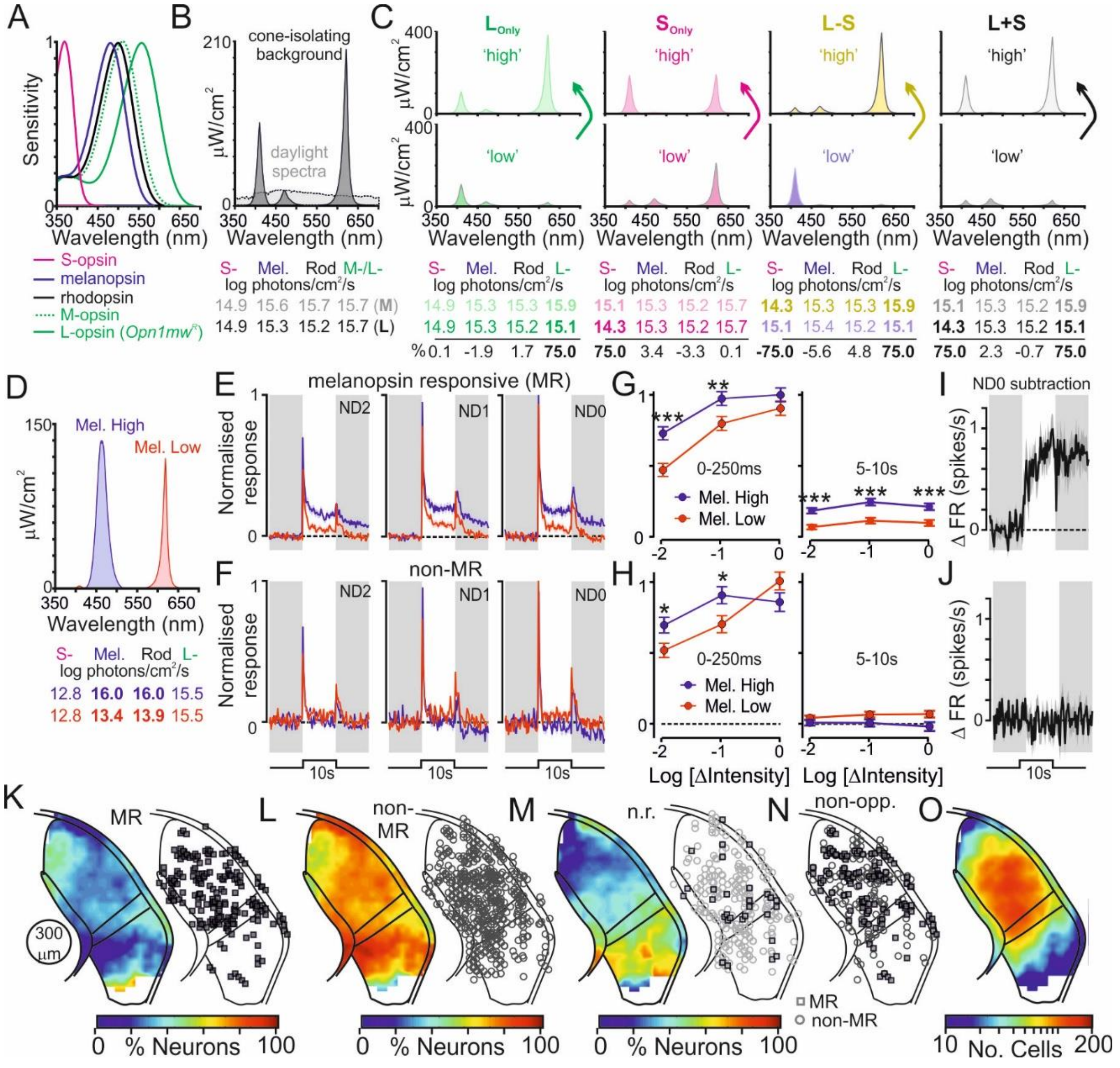

Figure S1. Stimuli used for selective modulation of cone responses and identification of melanopsin responsive neurons. Related to Figure 1. (**A**) Spectral sensitivity of mouse cone opsins, including the long-wavelength shifted opsin in *Opn1mw*^{R} mice. (**B**) Spectra and quantification of the daylight background used for *Opn1mw*^{R} LGN recordings and comparison with a natural daylight spectra (quantified according to cone sensitivity in wildtype mice). (**C**) Spectra and quantification for stimulus pairs designed to provide selective contrast (75% Michelson) for L- and/or S-cone opsin, transitions between spectra in the lower and upper panels respectively constitute (left to right): 'L_{Only}', 'S_{Only}', 'L+S-' and 'L+S+' stimuli. (**D**) Spectra of cone-matched, melanopsin high and low stimuli, with quantification for effective irradiance as above. (**E,F**) Mean±SEM normalised change in firing evoked by Mel. High and Low stimuli presented at 0.01, 0.1 or 1x intensity shown in **D** (ND2-0), for cells categorised as melanopsin responsive (MR; **E**, n=195) or non-MR (**F**, n=494). (**G**,**H**) Mean±SEM normalised response evoked by Mel. High and Low stimuli for MR (**G**) and non-MR (**H**) cells, as a function of light intensity during the first 250ms (left) or last 5s (right) of the light step. Data analysed by mixed-effects linear model with Sidak's post-tests ($\mathbf{G}_{Left}$: Stim.-$F_{1,917}$=20.47, P<0.001, Int.-$F_{2,780}$=34.1, P<0.001, Stim.XInt.- $F_{2,780}$=1.8, P=0.16; $\mathbf{G}_{Right}$: Stim.-$F_{1,1072}$=51.9, P<0.001, Int.- $F_{2,797}$=3.4, P=0.03, Stim.XInt.- $F_{2,797}$=0.19, P=0.83; $\mathbf{H}_{Left}$: Stim.-$F_{1,2873}$=2.8, P=0.1, Int.-$F_{2,2021}$=16.9, P<0.001, Stim.XInt.- $F_{2,2021}$=5.48, P=0.004; $\mathbf{H}_{Right}$: Stim.-$F_{1,2457}$=9.8, P=0.002, Int.-$F_{2,2071}$=0.13, P=0.88, Stim.XInt.- $F_{2,2071}$=0.82,P=0.44). (**I,J**) Mean±SEM difference in firing to Mel. High minus Mel. Low at ND0 for MR (**I**) and non-MR (**J**) cells. (**K,L**) Proportions MR (**K**) vs. non-MR (**L**) LGN neurons as a function of anatomical location (left, binned with 150μm radius window) and corresponding unit locations (right). (**M,N**) Anatomical distribution of LGN neurons (MR

and non-MR combined) that lacked response to cone isolating stimuli (**M**; n.r.) or exhibited non-opponent responses (**N**; corresponding heatmap shown in Figure 1J). (**O**) Overall density of cells used for spatial binning in Figure 1 and S1 (note: log scale).

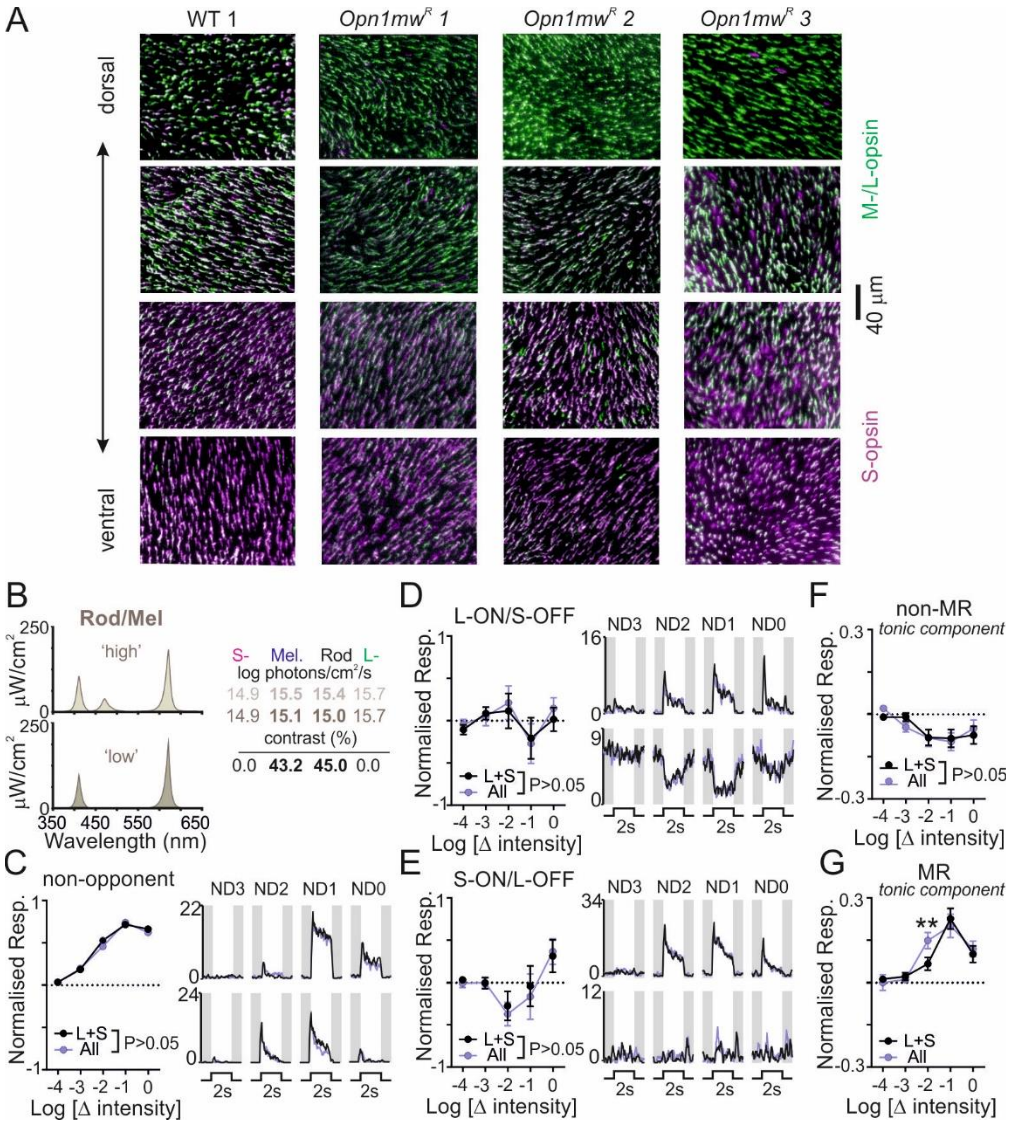

Figure S2. Opsin expression and cone-based response validation in red cone mice. Related to Figure 2. (**A**) Immunohistochemical detection of M-/L- and S-opsin expression across dorsal-ventral portions of retinas from wildtype (WT; left) and *Opn1mw*^{R} mice (mid-right). (**B**) Spectra and quantification for stimulus pairs designed to provide cone-silent contrast for rods and melanopsin (45% rod contrast). (**C-E**) Mean±SEM normalised firing responses (left) and responses of representative neurons (right; in each case upper row shows MR and lower row non-MR neuron) to 75% contrast stimuli modulating both cone types independent of, or concomitant with, changes in rod and melanopsin excitation (right panels; L+S vs. 'All' respectively) at varying background light intensities. Data derives from same neurons in Figure 2D-F (**C**: non-opponent; n=97, responses of n=16 OFF cells sign inverted; **D**: L-ON/S-OFF, n=14; **E**: S-ON/L-OFF, n=13). Data analysed by mixed-effects linear model (**C**: Stim.-$F_{1,884}$=0.6, P=0.45, Int.- $F_{4,408}$=211.3, P<0.001, Stim.XInt.- $F_{4,408}$=0.67, P=0.62; **D**: Stim.-$F_{1,79}$=0.14, P=0.71, Int.- $F_{4,37}$=2.0, P=0.11, Stim.XInt.- $F_{4,37}$=0.2, P=0.94; **E**: Stim.-$F_{1,65}$=0.2, P=0.68, Int.- $F_{4,31}$=4.2, P=0.008, Stim.XInt.- $F_{4,32}$=0.1, P=0.94). (**F-G**) Mean±SEM tonic modulation in firing (last 400ms at high and low stimulus phases) normalised to peak phasic response for non-MR (**F**; n=94; combined data from 71 non-opponent, 13 L-ON and 10 S-ON) and MR units (**G**; n=30; combined data from 26 non-opponent, 1 L-ON and 3 S-ON). Data analysed by mixed-effects linear model with Sidak's post-tests (**F**: Stim.-$F_{1,688}$=0.0, P=0.96, Int.- $F_{4,397}$=9.0, P<0.001, Stim.XInt.- $F_{4,399}$=1.8, P=0.13; **G**: Stim.-$F_{1,202}$=0.4, P=0.54, Int.- $F_{4,123}$=19.7, P<0.001, Stim.XInt.- $F_{4,123}$=3.6, P=0.009). ** indicates P<0.01.

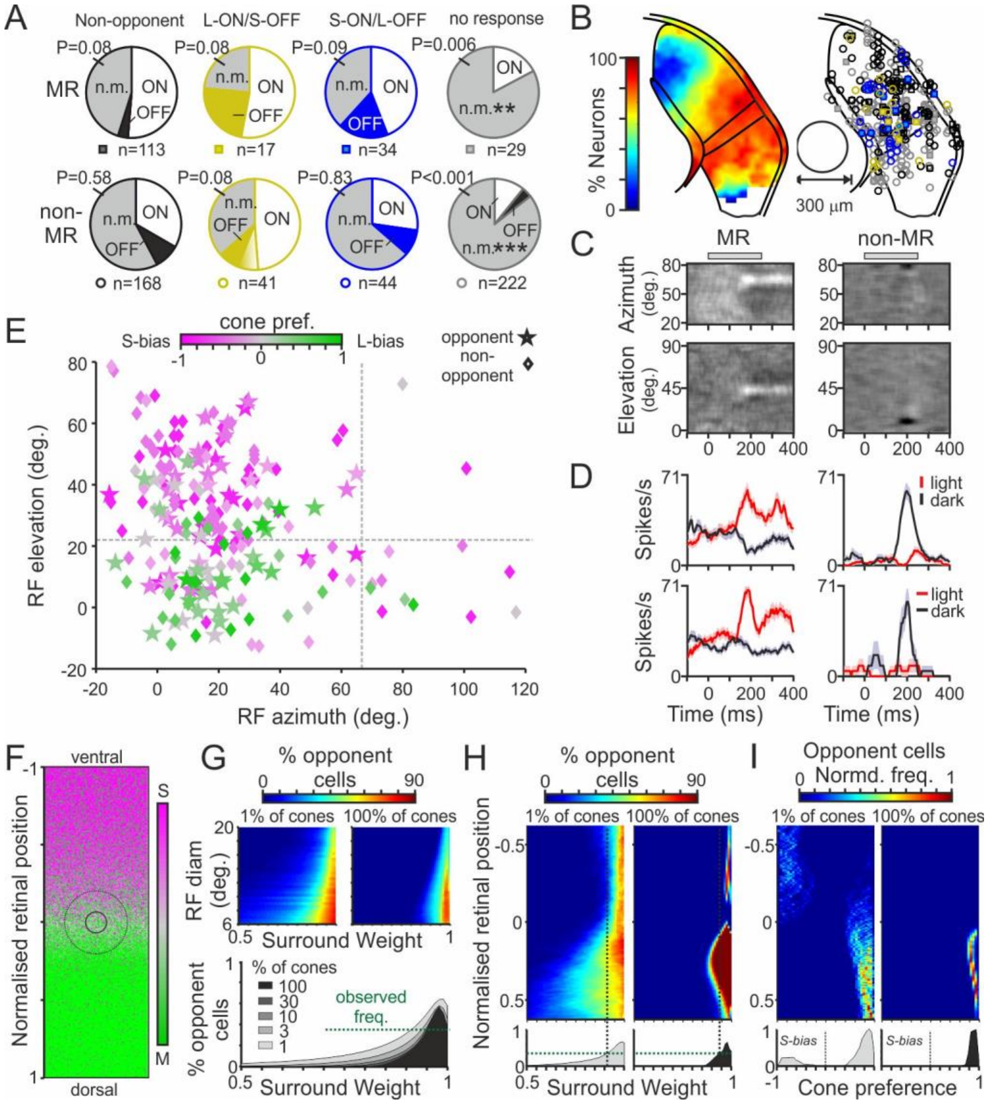

Figure S3. Receptive field positions and cone based responses. Related to Figure 3. (**A**) Distribution of RF properties (ON, OFF, ON/OFF or not mappable; n.m.) for MR and non-MR cells, subdivided based on responses to full field cone-isolating stimuli. Proportions of cells lacking mappable RFs were compared against that expected based on the proportion of visual field tested by Fishers exact test. (**B**) Left panels: proportions of neurons (n=92 MR and n=330 non-MR neurons) that lacked a mappable RF as a function of anatomical location (150µm radius binning), right panels: locations of individual cells. (**C**) Representative responses from MR (left) and non-MR (right) cells that lacked responses to full-field cone-isolating stimuli, conventions as in Figure 3A,B. (**D**) Mean±SEM firing rate following appearance of light and dark bars within the estimated RF centre for cells in **C**, conventions as in Figure 3C,D. (**E**) RF centre positions (defined relative to snout axis) for units in Figure 3E and F, colour coded according to cone preference from responses to full field cone-isolating stimuli (Figure 1). Dotted lines indicate projected midpoints of dorsal-ventral and nasal-temporal retinal axes. (**F-I**) Results from 'random-wiring' models of colour opponency (see Methods). (**F**) Example of modelled S- and M-cone opsin expression across a strip of retina and superimposed centre-surround RF profile (12°diameter, 36°surround). (**G**) Top panels show estimated percentages of opponent neurons as a function of RF diameter and surround weights from models where cells sampled from 1% (left) or 100% (right) of the cones within their RF. Lower panel shows the % of opponent neurons (averaged across all tested RF sizes) as a function of surround weight for cells that sampled randomly from varying proportions of cones. Dotted line shows the proportion of cells responding to full field stimuli with opponent responses from our data in Figure 1. (**H**) Estimated percentage of opponent neurons as a function of retinal position

and surround weight for 12°diameter RF cells (typical size for LGN neurons) sampling from 1% (left) or 100% (right) of cones within their RFs. Lower panels show average across the dorsal-ventral retina, with dotted lines indicating the surround weight producing a proportion of opponent cells that matches the experimental data. (**I**) Normalised distribution of cone preference as a function of retinal position for models that best matched observed proportion of opponent vs. non-opponent neurons in Figure 1 (12°diameter RF cells with surround weight = 0.91 and 0.94 in left and right panels; indicated by dotted lines in H).

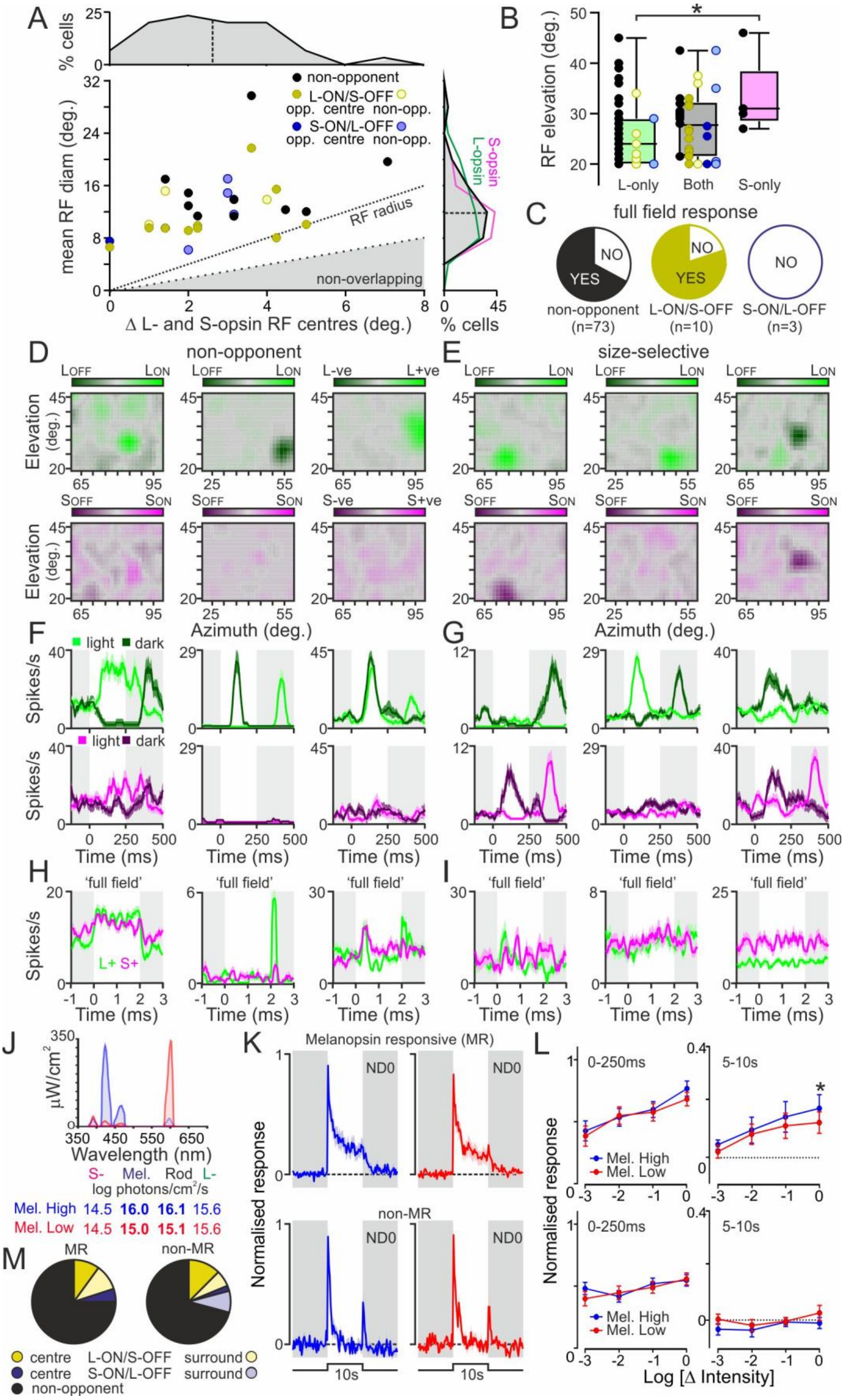

Figure S4. Properties of cone-dependent receptive fields in LGN neurons. Related to Figure 4. (**A**) Relationship between RF size and difference in apparent centre of L- and S-cone opsin RFs (for n=30 cells with mappable RFs for both). (**B**) Distribution of RF positions on elevation axis for neurons with mappable RFs to L- and/or S-opsin directed flashing squares. Data analysed by one-way ANOVA with Dunnett's post-tests ($F_{2,99}$=4.8, P=0.01). (**C**) Proportions of neurons with mappable cone-specific RFs that also responded to

full-field cone-specific stimulation. (**D**-**E**) L- and S-cone opsin dependent RF profiles for representative non-opponent neurons that did (**D**) or did not (**E**) exhibit robust responses to full field stimuli. (**F**-**G**) Mean±SEM responses to light and dark, L- and S-opsin directed squares (upper and lower panels respectively) appearing in the RF centre for neurons in **D**-**E**. (**H**-**I**) Mean±SEM responses to full-field 0.25Hz L- and S-cone opsin directed modulations for neurons in **D**-**G**. (**J**) Spectral power distribution of Mel. High and Mel. Low stimuli used to classify neurons as MR or non-MR. (**K**) Mean±SEM normalised firing rate profiles of MR (upper; n=20) and non-MR (lower, n=48) units presented with Mel. High and Mel. Low stimuli at maximum intensity. (**L**) Mean±SEM normalised responses of MR and non-MR units (as in **K**) during initial (left; first 250ms) and late (right; last 5s) of the 10s light steps. Data analysed by two-way RM ANOVA with Sidak's post-tests ($\mathbf{L}_{TopLeft}$: Stim.-$F_{1,19}$=1.2, P=0.28, Int.- $F_{3,57}$=5.1, P=0.004, Stim.XInt.- $F_{3,57}$=0.6, P=0.61; $\mathbf{L}_{TopRight}$: Stim.-$F_{1,19}$=8.7, P=0.008, Int.- $F_{3,57}$=2.7, P=0.05, Stim.XInt.- $F_{3,57}$=0.7, P=0.55; $\mathbf{L}_{BottomLeft}$: Stim.-$F_{1,47}$=0.4, P=0.53, Int.-$F_{3,141}$=2.5, P=0.06, Stim.XInt.- $F_{3,141}$=1.0, P=0.39; $\mathbf{L}_{BottomRight}$: Stim.-$F_{1,47}$=0.9, P=0.47, Int.-$F_{3,141}$=7.0, P=0.01, Stim.XInt.- $F_{3,141}$=1.5,P=0.21). * indicates P<0.05. (**M**) Classification of RF type for identified MR and non-MR neurons.
