# Supplementary material for: Extensive cone-dependent spectral opponency within a discrete zone of the lateral geniculate nucleus supporting mouse color vision
Source: Curr Biol. 2021 Aug 9;31(15):3391–3400.e4. doi: 10.1016/j.cub.2021.05.024 (PMC8360768; doi:10.1016/j.cub.2021.05.024)
Supplement: Document S2. Article plus supplemental information [file mmc2.pdf]

Report

Current Biology

Extensive cone-dependent spectral opponency within a discrete zone of the lateral geniculate nucleus supporting mouse color vision

Highlights

- Color opponency is common among neurons within a chromatic zone in the mouse LGN
- Cone inputs to LGN drive color opponency across mesopic to photopic light levels
- Cone inputs to opponent neurons derive from central and upper visual field
- Subsets of LGN neurons display opponency for small or large stimuli only

Authors

Josh W. Mouland, Abigail Pienaar, Christopher Williams, Alex J. Watson, Robert J. Lucas, Timothy M. Brown

Correspondence

timothy.brown@manchester.ac.uk

In brief

Mouland et al. examine color processing in the mouse visual thalamus. They show that color opponency is widespread among cells within a specific zone of the lateral geniculate nucleus and that cones drive such responses, independent of cone-opsin expression gradients and rod input, providing a robust capacity for color discrimination in mice.

Check for updates

Mouland et al., 2021, Current Biology *31*, 3391–3400
August 9, 2021 © 2021 The Authors. Published by Elsevier Inc.
https://doi.org/10.1016/j.cub.2021.05.024

CellPress

Current Biology

CellPress
OPEN ACCESS

Report

Extensive cone-dependent spectral opponency within a discrete zone of the lateral geniculate nucleus supporting mouse color vision

Josh W. Mouland,[1] Abigail Pienaar,[1] Christopher Williams,[1] Alex J. Watson,[1] Robert J. Lucas,[1] and Timothy M. Brown[1,2,3,*]
[1]Centre for Biological Timing, Faculty of Biology, Medicine and Health, University of Manchester, Manchester M13 9PT, UK
[2]Twitter: @BrownlabV
[3]Lead contact
*Correspondence: timothy.brown@manchester.ac.uk
https://doi.org/10.1016/j.cub.2021.05.024

SUMMARY

Color vision, originating with opponent processing of spectrally distinct photoreceptor signals, plays important roles in animal behavior.[1–4] Surprisingly, however, comparatively little is understood about color processing in the brain, including in widely used laboratory mammals such as mice. The retinal gradient in S- and M-cone opsin (co-)expression has traditionally been considered an impediment to mouse color vision.[5–8] However, recent data indicate that mice exhibit robust chromatic discrimination within the central-upper visual field.[9] Retinal color opponency has been reported to emerge from superimposing inhibitory surround receptive fields on the cone opsin expression gradient, and by introducing opponent rod signals in retinal regions with sparse M-cone opsin expression.[10–13] The relative importance of these proposed mechanisms in determining the properties of neurons at higher visual processing stages remains unknown. We address these questions using multielectrode recordings from the lateral geniculate nucleus (LGN) in mice with altered M-cone spectral sensitivity (*Opn1mw^R*) and multispectral stimuli that allow selective modulation of signaling by individual opsin classes. Remarkably, we find many (~25%) LGN cells are color opponent, that such cells are localized to a distinct medial LGN zone and that their properties cannot simply be explained by the proposed retinal opponent mechanisms. Opponent responses in LGN can be driven solely by cones, independent of cone-opsin expression gradients and rod input, with many cells exhibiting spatially congruent antagonistic receptive fields. Our data therefore suggest previously unidentified mechanisms may support extensive and sophisticated color processing in the mouse LGN.

RESULTS AND DISCUSSION

Extensive color opponency in mouse LGN

Established mechanisms of mammalian color discrimination involve opponent processing of cone photoreceptor signals.[3,4] Like most mammals, mice possess two cone opsins (S- and M-opsin, maximally sensitive in UV and "green" portions of the spectrum, respectively; Figure S1A) that could theoretically support color vision. The extent to which cone opponency forms a major component of mouse vision remains unclear, however. Several studies have reported opponent responses to UV and "green" light (in some cases under adapting backgrounds) in subsets of retinal ganglion cells (RGCs), lateral geniculate nucleus (LGN), and visual cortex neurons.[10–16] However, while the resulting responses are often interpreted as originating with S-opsin and M-opsin, respectively, all opsins exhibit reasonable UV sensitivity (including *in vivo* for species like mice with UV-transmitting lenses); thus, UV responses can occur even with no involvement of S-opsin.[17,18] Further, the close spectral sensitivity of mouse M-opsin and rhodopsin (Figure S1A) makes the separating rod/cone contributions to "green"-biased challenging, especially since rod responses can persist even under high background light levels following sufficient adaptation.[19] Indeed, the recent observation of opponent responses in many RGCs in ventral retina,[13] where most cones express S-opsin, has been taken as evidence for a significant role of rods in chromatic opponency.

We set out to unambiguously determine the extent to which cone-opponent mechanisms account for color vision in mice by performing multielectrode recordings spanning the visual thalamus in mice (n = 27) in which the native M-cone opsin was replaced with the human L-cone opsin[20] (*Opn1mw^R*; Figure S1A). We then employed previously validated[21] multispectral stimuli to selectively modulate effective light intensity for L- and/or S-cone opsin, against a background that recreated a wild-type mouse's experience of natural daylight (Figures S1B and S1C). Using these stimuli, cone-opponent responses are readily identifiable via opposite responses to selective modulation of either cone opsin ("L_{Only}" and "S_{Only}" stimuli) and via stronger responses to antiphasic ("L-S," producing a substantial change in color) rather than in synchronous changes in excitation of both cone opsins ("L+S," which changes "illuminance" without altering color).

This is an open access article under the CC BY license (http://creativecommons.org/licenses/by/4.0/).

A sizeable proportion of LGN neurons (n = 433/715) responded to these stimuli, presented as full-field square wave modulations across a range of contrasts (Figures 1A–1D). Many of these (n = 283) were not color opponent, showing responses of the same sign to L_{Only} and S_{Only} stimuli (Figures 1A and 1B), albeit often with a strong bias toward one stimulus (Figures 1E and 1F). Combined stimulation of both opsins confirmed this lack of opponency, as L+S stimuli produced modestly (but significantly) larger modulations in firing than the L-S stimulus (Figure 1E). However, the remaining 150 LGN neurons (~35% of responsive cells) displayed either L-ON/S-OFF (Figure 1C; n = 72) or S-ON/L-OFF (Figure 1D; n = 78) opponency, evidenced by responses of opposite sign to L_{Only} and S_{Only} stimuli and substantially larger responses to chromatic L-S modulations versus achromatic L+S modulation (Figure 1G). Hence, while the relative amplitude of L_{Only} and S_{Only} responses varied across opponent neurons, these were usually less skewed than for non-opponent cells (Figures 1G and 1H).

Anatomical specialization of color processing in the mouse LGN

We next asked how these responses mapped to the known topographic arrangements[22] and subdivisions of the LGN complex (i.e., dorsal LGN, which provides main cortical relay, and intergeniculate leaflet and ventral LGN, which are involved in circadian and accessory visual functions; dLGN, IGL, and vLGN, respectively). Across all cone-responsive cells, the relative amplitude of L_{Only} versus S_{Only} responses varied strongly with anatomical location, with equal preference in medial portions of the dLGN and IGL, strong S-opsin preference in dLGN and lateral vLGN, and strong L-opsin preference in medial vLGN (Figure 1I). This arrangement indicates a robust topographic ordering of S-opsin-rich ventral and L-opsin-rich dorsal retinal output across the LGN complex.

We then examined the prevalence of cells exhibiting non-opponent and opponent responses, revealing a pronounced functional subdivision where opponent neurons strongly localized to medial dLGN and IGL and non-opponent neurons dominated the surrounding LGN regions (Figure 1J). Hence, we identify a clear color-opponent zone within the mouse LGN. This region strongly overlaps with LGN innervation by melanopsin-expressing RGCs (mRGCs)[23,24] of which M5[12] and potentially also M4[10,25] subtypes could drive color opponency. Accordingly, we investigated the extent to which color opponency overlapped melanopsin-driven responses ("melanopsin-responsive"; MR neurons).

To this end, we employed a validated strategy[21] of comparing responses to "Mel. High" and "Mel. Low" light steps (10 s) matched for activation of both cone opsins but differing ~500-fold in effective irradiance for melanopsin (Figure S1D). Since the stimuli also differed in their impact on rods, they were presented across an intensity range that drives transient rod saturation even for the Mel. Low stimulus (11.9–13.9 rod effective photons/cm^2/s). Many LGN neurons responded to these stimuli (n = 689/715, including cells that lacked robust responses to cone-isolating stimuli), with a subset (n = 195) displaying differential responses indicative of melanopsin input (Figures S1E, S1G, and S1I). Specifically, in keeping with the sluggish kinetics of melanopsin phototransduction,[26] at the highest intensities the initial increase in firing (first 250 ms) for MR units was equivalent for both stimuli, while sustained increases in firing (last 5 s) were reliably greater for Mel. High (Figures S1G and S1H). By contrast, among the remaining "non-MR" cells, tonic responses to Mel. High never exceed those for Mel. Low (Figure S1F, S1H, and S1J). The prevalence and properties of MR cells matched those detected in the LGN using other approaches,[24,27–29] and their anatomical localization aligned with the known distribution of mRGC projections[23,24] across the IGL/vLGN and around the medial border of the dLGN (Figures S1K and S1L).

Cross-referencing against cone-driven responses revealed that a subset of color-opponent cells was also MR (n = 34/78 S-ON/L-OFF and n = 19/72 L-ON/S-OFF cells). However, an equivalent subset of non-opponent cells was also MR (n = 113/283); thus, the presence of melanopsin input was not predictive of the occurrence or nature of color opponency (χ^2 test, p = 0.06). There were also no major differences in the anatomical locations (Figure 1K) or properties of MR and non-MR opponent responses (Figure 1L). MR neurons were, however, substantially more likely to respond to cone-isolating stimuli overall (Figure 1M). The relatively high proportion of non-MR cells that did not respond (n = 227) presumably reflects a low sensitivity to full field contrast in those cells that were especially prevalent in dorsolateral dLGN and medial vLGN (Figures S1M and S1N), regions known to receive input from direction-selective RGCs.[30,31]

Rods or melanopsin cannot account for LGN color opponency

We next confirmed that cone-opsin expression was not abnormal in *Opn1mw*^{R} mice. Consistent with previous reports that L-opsin expression in such animals faithfully recapitulates the native M-opsin expression,[19] retinas from *Opn1mw*^{R} mice had normal gradients in S- and L-opsin (co)-expression equivalent to wild-type animals[5–7] (Figure S2A).

Since rods may function at surprisingly high light levels,[18] we also aimed to rule out rod contributions. Nominal rod contrast for our cone-isolating stimuli is negligible (Figure S1C). To directly test rod contributions, we evaluated responses to a stimulus designed to provide 45% contrast from rods but <0.05% contrast for cones (Figure S1B). Across cell groups (Figures 2A–2C), responses to this "Rod" stimulus were undetectable, whereas 45% contrast cone-modulating stimuli reliably evoked significant changes in firing, consistent with properties reported above.

In some experiments, we further assessed the stability of cone-driven responses across five logarithmically spaced background light levels (ND4–ND0) spanning the mesopic to photopic range. As expected, responses to L_{Only} and S_{Only} stimuli (75% contrast) were negligible at ND4, typically becoming detectable at ND3 and robustly present across all cell groups at ND2–ND0 with the expected polarity (Figures 2D–2F). To probe the possibility that rods might also contribute to opponency in the LGN at these lower background light levels, we further compared responses to 75% contrast L+S stimulus with a contrast-matched stimulus targeting all photoreceptors ("All-opsin"). If rods were contributing to color opponency, responses to the latter should differ from the cone-selective L+S stimulus. In fact, there were no detectable differences in response (Figures S2C–S2E), even at the dimmest background, where very few cells responded

CellPress
OPEN ACCESS

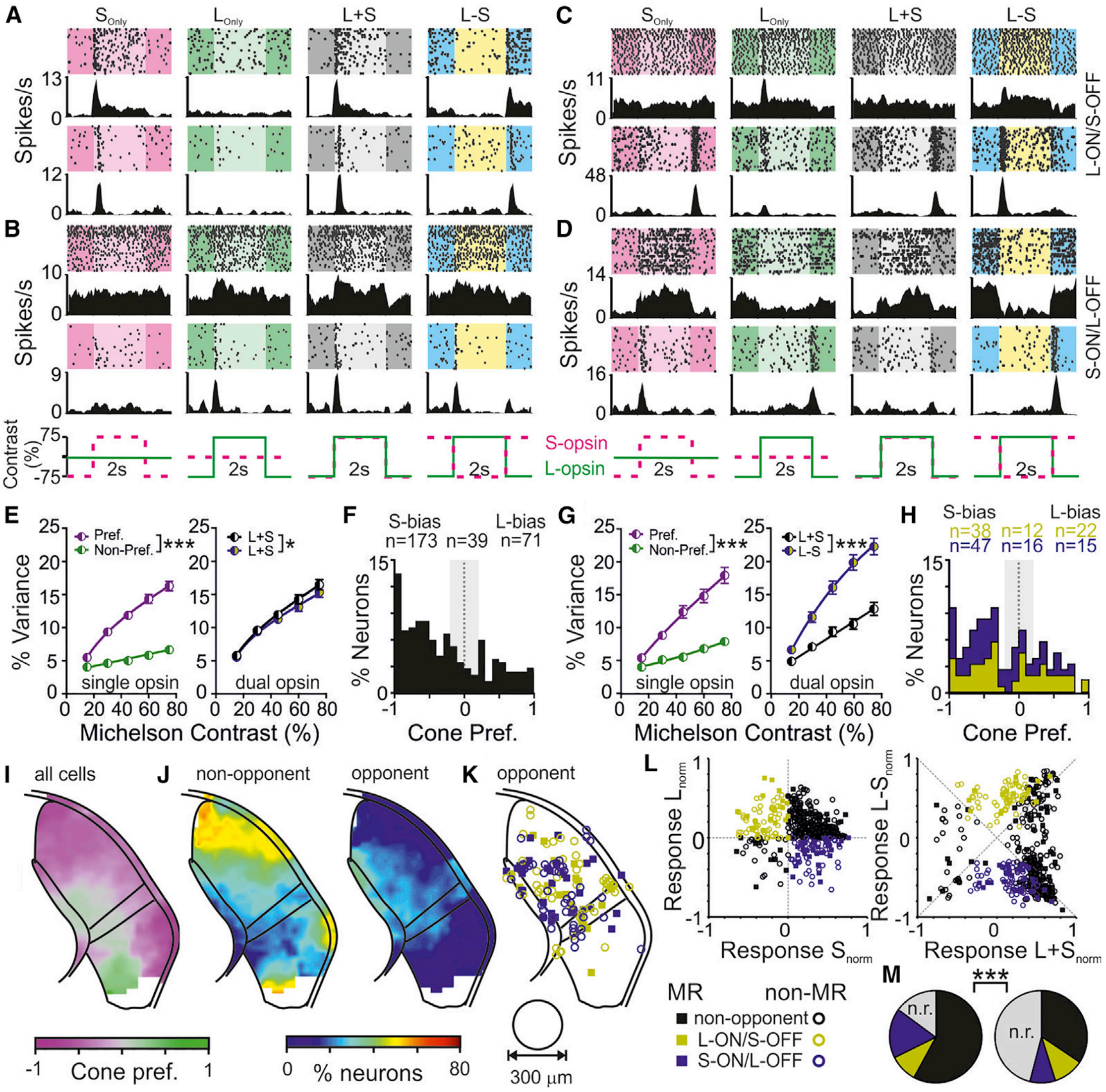

Figure 1. Cone-dependent color opponency is enriched across medial LGN neurons

(A–D) Responses to 75% contrast cone-isolating stimuli (spike rasters and corresponding histograms) from two representative neurons (each neuron on a different horizontal row) classified as non-opponent, S- (A) or L-opsin biased (B), and opponent L-ON/S-OFF (C) or S-ON/L-OFF (D).

(E) Mean ± SEM contrast response relationships for single (left; sorted according to preferred versus non-preferred opsin) and dual cone opsin stimulation (right; L+S versus L-S) for non-opponent LGN neurons (n = 283). Data analyzed by mixed-effects linear model (left: Stim., $F_{1,1360} = 553.5$, $p < 0.001$; Con., $F_{4,682} = 128.0$, $p < 0.001$; Stim. × Con., $F_{4,682} = 56.7$, $p < 0.001$; right: Stim., $F_{1,2085} = 3.9$, $p = 0.048$; Con., $F_{4,1003} = 180.1$, $p < 0.001$; Stim. × Con., $F_{4,1003} = 0.4$, $p = 0.78$).

(F) Distribution of cone opsin preference ($[L_R - S_R]/[L_R + S_R]$) for non-opponent LGN neurons; shading indicates cells with well-matched responses to both opsins (<50% difference in response amplitude).

(G) As in (E) but for opponent LGN neurons (n = 150; left: Stim., $F_{1,605} = 216.6$, $p < 0.001$; Con., $F_{4,336} = 75.2$, $p < 0.001$; Stim. × Con., $F_{4,422} = 2.3$, $p = 0.06$; right: Stim., $F_{1,952} = 166.0$, $p < 0.001$; Con., $F_{4,477} = 112.0$, $p < 0.001$; Stim. × Con., $F_{4,336} = 23.0$, $p < 0.001$).

(H) As (F) but for opponent LGN neurons.

(I) Mean cone preference for LGN neuron populations in (F) and (H) relative to anatomical location in LGN (150 μm radius moving window).

(J) Prevalence of non-opponent (left) versus opponent (right) neurons as a function of anatomical location in the LGN; proportion expressed relative to total number of neurons (including LGN cells that did not respond to cone-isolating stimuli; data for sampling density presented in Figure S1O).

(K) Projected anatomical locations of opponent neurons (corresponding to right panel in J) with classification as melanopsin responsive (MR) versus non-MR.

(L) Normalized firing responses across all neurons contributing to (E)–(K) for S_{Only} versus L_{Only} (left) and L+S versus L-S stimuli (right) subdivided according to cell classification.

(M) Proportions of LGN cells with non-opponent, opponent, or no response (n.r.) to cone-isolating stimuli for MR (left) and non-MR groups (right; χ^2 test; $\chi^2 = 64.3$, df = 3, $p < 0.001$).

*p < 0.05, **p < 0.01, and ***p < 0.001. See also Figure S1 for details of stimuli used in (A)–(H) and data related to cell classification as MR/non-MR in (K)–(M).

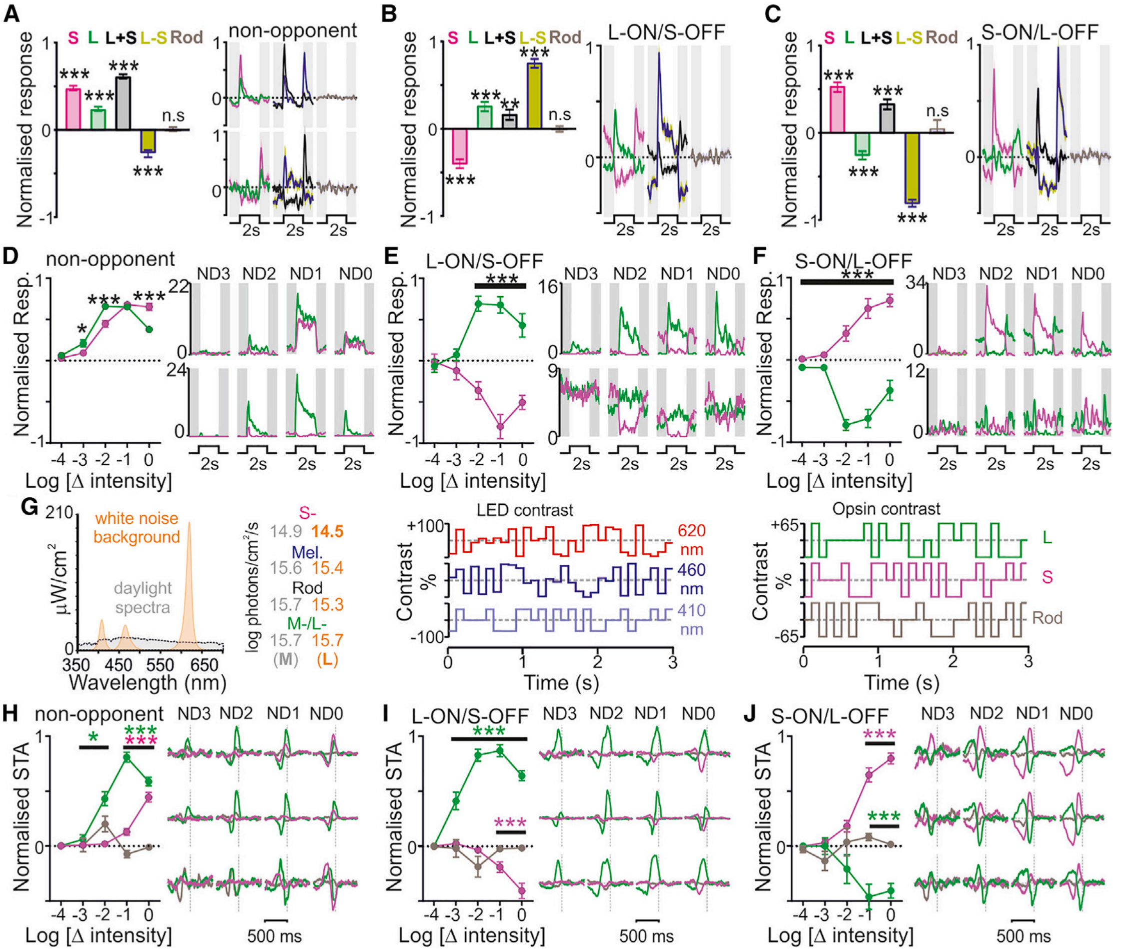

Figure 2. Rod signals do not play a major role in driving LGN neuronal responses across photopic light levels

(A–C) Left panels show normalized mean ± SEM responses of non-opponent (A; n = 283, responses of n = 31 OFF cells sign inverted), L-ON/S-OFF (B; n = 72), and S-ON/L-OFF (C; n = 78) LGN neurons to 45% contrast cone-modulating stimuli and a cone-silent 45% rod contrast stimulus. Data analyzed by one-sample t tests versus hypothetical value of zero. Right panels show normalized mean ± SEM response profiles for each group of neurons (ON and OFF non-opponent responses in A plotted separately).

(D–F) Left panels show mean ± SEM normalized firing responses to 75% contrast L- and S-cone modulating stimuli at varying background light intensities (ND4–ND0) for non-opponent (D; n = 97, responses of n = 16 OFF cells sign inverted), L-ON/S-OFF (E; n = 14), and S-ON/L-OFF (F; n = 13) LGN neurons. Data analyzed by mixed-effects linear model with Sidak's post-tests (D, Stim., $F_{1,881} = 0.3$, $p = 0.59$; Int., $F_{4,351} = 132.7$, $p < 0.001$, Stim. × Int., $F_{4,351} = 11.6$, $p < 0.001$; E, Stim., $F_{1,106} = 117.3$, $p < 0.001$; Int., $F_{4,51} = 1.7$, $p = 0.17$; Stim. × Int., $F_{4,51} = 19.4$, $p < 0.001$; F, Stim., $F_{1,70} = 225.8$, $p < 0.001$; Int., $F_{4,36} = 5.5$, $p = 0.002$; Stim. × Int., $F_{4,36} = 38.1$, $p < 0.001$). Right panels show firing responses (spikes/s) of two representative neurons of each class.

(G) Left: spectral composition of background stimulus (with reduced S-cone irradiance relative to natural daylight) used for white noise experiments dissociating cone and rod signals. Middle and right panels show a segment of a 10 Hz white noise stimulus illustrating modulation of the LED sources and resulting contrast for L-, S-, and rod opsin (±65% contrast relative to background).

(H–J) Left panels show mean ± SEM normalized amplitude of spike-triggered averages (STAs) of the white noise stimulus at varying background light intensities (ND4–ND0) for non-opponent (H; n = 72, responses of n = 10 OFF cells sign inverted), L-ON/S-OFF (I; n = 23), and S-ON/L-OFF (J; n = 25) LGN neurons. Data analyzed by mixed-effects linear model with Sidak's post-tests against Rod STA (H, Stim., $F_{2,4018} = 4.5$, $p = 0.03$; Int., $F_{4,374} = 165.3$, $p < 0.001$; Stim. × Int., $F_{8,283} = 82.9$, $p < 0.001$; I, Stim., $F_{2,98} = 266.1$, $p < 0.001$; Int., $F_{4,97} = 31.6$, $p < 0.001$; Stim. × Int., $F_{8,89} = 92.9$, $p < 0.001$; J, Stim., $F_{2,143} = 92.0$, $p < 0.001$; Int., $F_{4,133} = 6.9$, $p < 0.001$; Stim. × Int., $F_{8,115} = 41.3$, $p < 0.001$). Right panels show mean ± SEM opsin-specific STAs for three representative neurons of each class. *p < 0.05, **p < 0.01, and ***p < 0.001. See also Figure S2 for additional control data on cone opsin expression and rod-based responses tested in (A)–(F).

to either stimulus. The latter observation presumably reflects a retinal adaptation state where cone responses are minimal and rods are transiently saturated (testing completed within 10 min of stepping from darkness to ND4). While not excluding the possibility that rod opponency may become more apparent following more extensive adaptation at mesopic light levels, these data confirm that, as expected under our experimental conditions,[19,21,32] LGN responses are overwhelming cone, not rod, driven.

As the All-opsin stimulus also provided contrast for melanopsin, it further allowed us to explore suggestions that melanopsin might contribute to color opponency.[4] Although the 0.25 Hz modulations employed are not optimal for eliciting melanopsin responses, there was a small difference in sustained response

(last 400 ms at each stimulus phase) between L+S and All-opsin responses at moderate intensities for cells categorized as MR (Figure S2G), but not non-MR (Figure S2F). Nevertheless, the strong similarity between responses to these two stimuli even in MR cells (Figures S2C–S2E) argues that any melanopsin contribution to the color opponent signal is minimal under these conditions.

Returning to the role of rods, we additionally investigate the possibility that some feature of the experimental design (e.g., high contrast nature of stimuli) might mask rod contributions. Accordingly, we designed a white noise stimulus that allowed us to independently modulate rod and cone contrast. Since our original background stimulus (Figure S1B) substantially curtailed the degree to which this was achievable, we designed a new background with a reduced S-opsin irradiance relative to natural daylight (Figure 2G). We then presented pseudorandom (±65%, 10 Hz) contrast sequences (Figure 2G) across a range of light levels (ND4–ND0).

Consistent with data above, spike-triggered averages of rod and cone components of white noise stimulus were negligible at ND4 (Figures 2H–2J). Cone-driven responses became detectable in some cells at ND3 and were consistently detected at the highest two intensities. While the reduced S-opsin irradiance of the background rendered responses more L-opsin biased than observed using our previous stimulus set, the polarity of cone responses was consistent with their classification as opponent or non-opponent using those other stimuli (Figure 1). We did also, in some cases, detect weak rod responses at dimmer backgrounds (ND3 and ND2), but these were absent at irradiance levels where we found robust S-cone-driven responses (Figures 2H–2J). Hence, again, we did not see direct evidence for significant rod contributions to chromatic discrimination under the conditions studied here (equivalent to natural light levels spanning civil twilight to daytime[33]).

LGN color opponency across the central visual field

Given reports that color discrimination may preferentially localize to the upper visual field in mice,[7,9,13,15] we next mapped spatial receptive fields (RFs) for cone-opponent LGN neurons. To allow for rapid estimation of RF position across substantial portions of the ~180° mouse field of view,[34] we initially used the fairly crude approach of presenting light or dark, horizontal or vertical bars, at varying visual locations across the central and upper visual field (STAR Methods). This allowed us to determine RF position for a sizeable subset of units, including many opponent and non-opponent neurons (Figures 3A–3D; n = 209/417 cone-responsive cells). Across groups, the proportions of neurons where we could map an RF was commensurate with the proportion of the visual field (~45%) tested (Figure S3A), with cells lacking a detectable RF strongly clustered within specific portions of the LGN complex (Figure S3B). We could also occasionally map RFs for cells that did not respond to full-field cone isolating stimuli (n = 34/228), although, in this case, with significantly lower frequency than expected (Figure S3A), suggesting that such cells may be more strongly tuned to stimulus size and/or other visual features (e.g., motion) that our stimuli did not provide.

Importantly, among cone-responsive cells we identified a clear relationship between RF position and opsin preference. Specifically, the relative magnitude of full-field S- versus L-opsin-driven responses varied strongly according to RF elevation (Figure 3E), but not azimuth (Figures 3F and S3E). Hence, portions of visual space associated with more ventral retinal locations exhibited increasing S-opsin bias, consistent with the known gradient of S-opsin expression.[6,15] By contrast, the prevalence of opponent neurons did not vary significantly according to retino-/visuotopic location (Figures 3E and 3F). Insofar as the vast majority of cells analyzed here had receptive fields located at elevations of −10° to 60° relative to the snout axis (i.e., the central ~70° of the mouse retina, accounting for the tilt of the eye), however, color-opponent neurons may be less common at the extremes of the visual field.

We next modeled the likelihood that the observed LGN color opponency could be an emergent property of asymmetries in cone contributions to center and surround RF components ("random wiring"; Figures S3F–S3I; STAR Methods). We found that the retinal gradients in mouse cone opsin expression could theoretically result in an equivalent proportion of opponent neurons as in our experimental data, but this was critically dependent on very strong surround suppression (Figure S3G). A further feature of such models was that opponent neurons should be substantially more common among cells with RFs dorsal as opposed to ventral to the midpoint of the retina (Figure S3H) and that the relative weighting of cone inputs to such opponent cells was very strongly biased toward L- or S-cone opsin, dependent on RF location (Figure S3I). These features do not align well with our experimental observations, strongly suggesting that cone subtype-selective retinal circuits (e.g., Nadal-Nicolás et al.,[7] Haverkamp et al.,[8] and Breuninger et al.[35]) play some role(s) in generating the chromatic responses of mouse LGN neurons.

Receptive field properties supporting color opponency in mouse LGN

While our modeling suggests a random-wiring mechanism is most unlikely to fully account for mouse LGN color opponency, there is certainly evidence that retinal color opponency can result from surround inhibition.[10–13] A feature of such mechanisms is that opponent responses are often only readily detectable for stimuli that extend beyond the RF center. To determine if this was also true for LGN color opponency, we investigated RF properties following selective stimulation of the two cone subtypes using a custom projector system that allowed us to spatially modulate effective radiance targeting just L- or S-opsin. We then evaluated responses to spatially localized increments and decrements in L- or S-opsin excitation appearing at random locations across the central visual field (Figure 4A; STAR Methods).

From recordings in 23 *Opn1mw*^{R} mice, we identified 102 LGN neurons where we could reliably map an RF using L- and/or S-opsin-directed spatial stimulation (Figures 4B–4F and S4A–S4E). Consistent with the strong bias in cone-based responses reported above, the majority of such cells only exhibited robust responses to stimulation of one cone opsin type (Figure 4B; n = 67, L-opsin only; n = 5, S-opsin only). This, at least partly, reflected the retinal gradient of cone opsin expression, since RFs for cells with pure S-driven responses were located at higher elevations than those with pure L-driven responses (Figure S4B).

Nonetheless, among the 30 neurons for which we could map RFs for both cone-types, L- and S-opsin-dependent RFs were

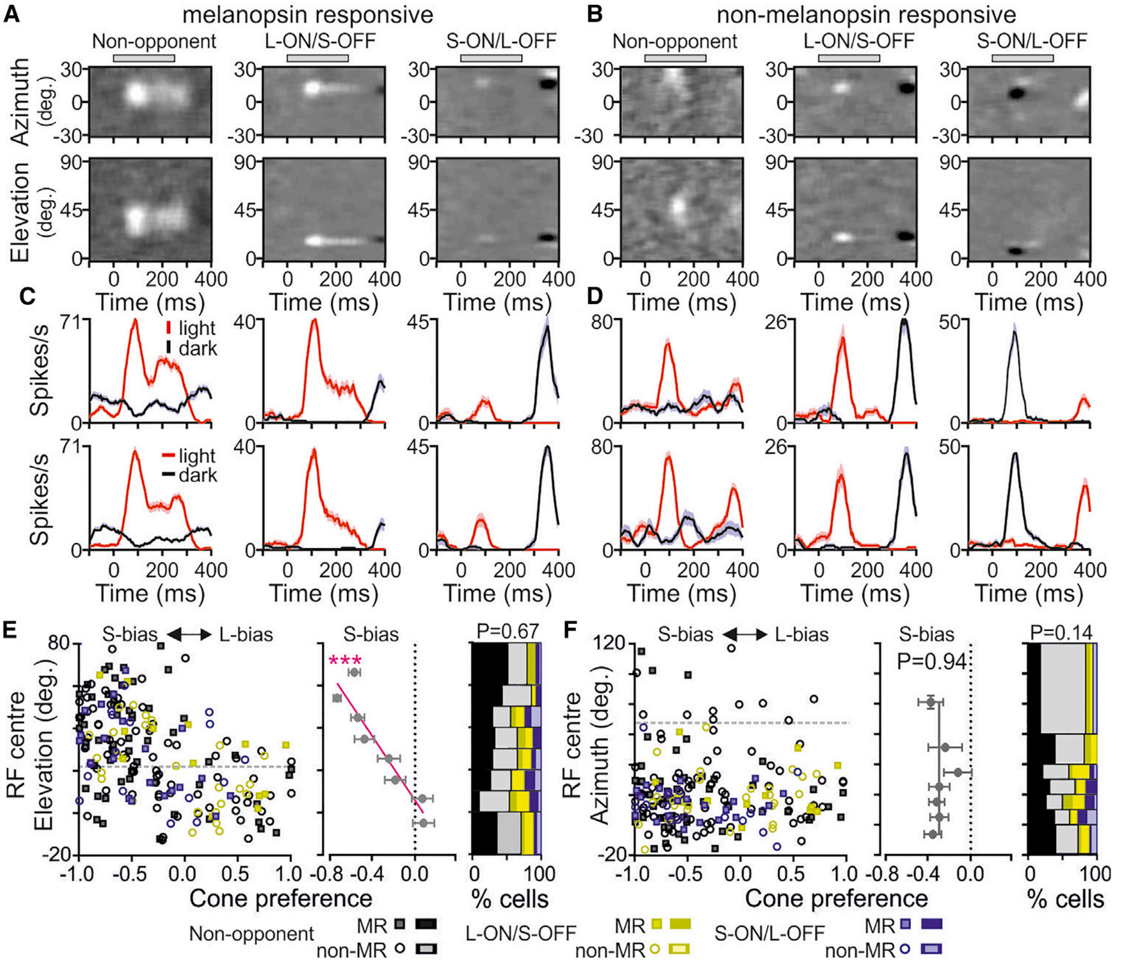

Figure 3. Relationship between receptive field location and cone-driven response properties of LGN neurons

(A and B) Representative responses from non-opponent (left) and opponent (middle and right) MR (A) and non-MR (B) cells, showing normalized difference in firing evoked by 250 ms appearance of light and dark horizontal (top) or vertical (bottom) bars at varying azimuth or elevation, respectively.

(C and D) Mean ± SEM firing rate following appearance of light and dark horizontal (top) or vertical (bottom) bars within the estimated RF center for cells shown in (A) and (B).

(E and F) Left panels show relationship between cone preference determined using full field cone isolating stimuli and RF position on elevation (E) or azimuth (F) axes. Center panels show mean ± SEM cone preference across all cell types binned according to RF positions with linear fit. Extra sum-of-squares F-test indicated the relationship between cone preference and RF elevation had a non-zero slope (E; $F_{1,6} = 45.6$, $p = 0.0005$) while that for RF azimuth did not (F; $F_{1,5} = 0.01$, $p = 0.94$). RF positions are defined relative to the axis of the mouse snout. Dotted horizontal lines indicate projected midpoints of dorsal-ventral (E) and nasal-temporal (F) retinal axes. Right panels show relative proportion of each cell type binned as above. Chi-square tests indicated proportions of each cell type did not vary as a function of RF position (E, $\chi^2 = 30.8$, df = 35, $p = 0.67$; F, $\chi^2 = 38.5$, df = 30, $p = 0.14$).

See also Figure S3 for additional data on spatial response properties for units and relationship to expected properties of opponent neurons predicted from random wiring models.

strongly overlapping (Figure S4A) and, in a subset of cases, L-ON/S-OFF (Figures 4D and 4G; n = 10) or S-ON/L-OFF (Figures 4E and 4H; n = 3) opponent. These observations suggest a different mechanism of chromatic processing to the surround-based mechanisms previously observed in RGCs, where cells usually lack detectable responses to small spots.[10–13] To probe for the latter, we also then compared the cone-driven RFs of all cells to their responses to equivalent full-field stimuli (Figures 4J–4L, S4H, and S4I). This revealed a further subset of cells that lacked opponent RFs but displayed either L-ON/S-OFF (n = 10) or S-ON/L-OFF (n = 6) opponency to full-field stimulation (Figures 4F, 4I, and 4L). In most other cases, responses to full-field cone isolating stimuli were consistent with the observable L- and/or S-opsin-driven RFs (Figures 4G, 4J, S3F, and S3H). Interestingly, however, cells with S-ON/L-OFF RFs lacked detectable responses to full-field stimuli (Figure 4K), suggesting strong surround suppression in this group. A similar property was found with reasonable frequency in cells with non-opponent (n = 24/73) RFs but was comparatively rare in cells with L-ON/S-OFF RFs (n = 10; Figures S4C, S4E, S4G, and S4I).

In total, then, a sizeable proportion of cells (>25%) exhibited color opponency, comprising a near-equal mixture of those where opponent responses were detectable for spatially discrete (n = 13) or only for full-field (n = 16) stimuli, potentially

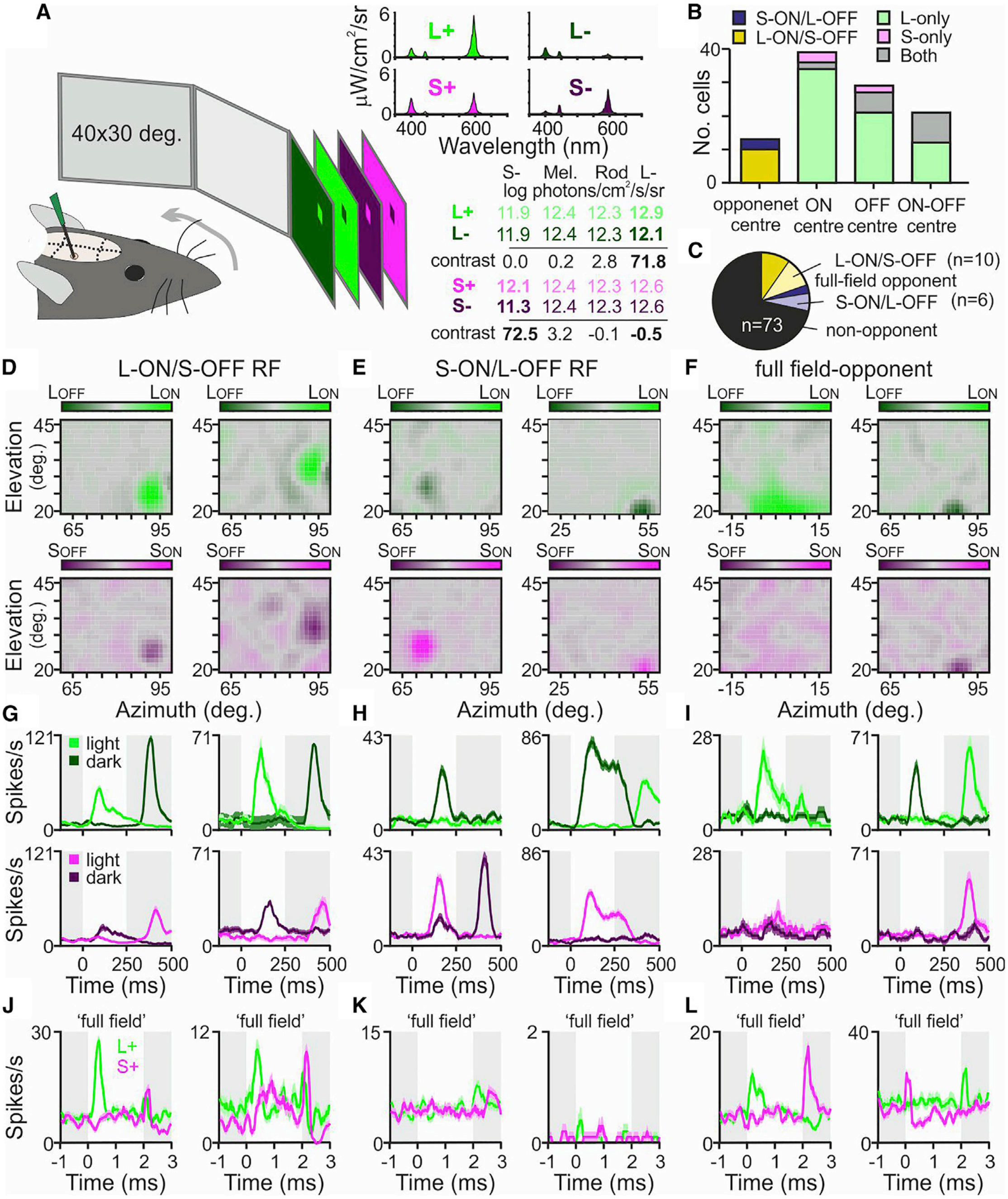

Figure 4. Color opponent receptive fields in subsets of LGN neurons

(A) Schematic of experimental setup for assessing L- and S-cone opsin-specific RFs with a multiprimary projector system; ~6° squares providing selective increments or decrements in radiance for L- or S-cone opsin presented at random, overlapping locations on a 40° × 30° display (72% Michelson contrast, 250 ms appearance) placed sequentially at 3 locations across the upper-central visual field. Note that elevations are specified relative to midpoint of snout axis; the projected midpoint of the dorsal-ventral retinal axis occurs at an elevation of 22°.

(B) Classifications of LGN neuronal responses to L- and/or S-cone opsin-modulating squares.

(C) Classification of RF type for LGN neurons based on spatially patterned and full-field stimuli, showing cells with opponent RFs and full-field opponency (darker and lighter shading, respectively) of each type and non-opponent neurons.

(D–F) L- and S-cone opsin-dependent RF profiles for representative neurons showing L-ON/S-OFF (D), S-ON/L-OFF (E), and non-opponent RFs, but opponent responses to widefield stimulation suggesting a surround-based opponent mechanism (F).

(G–I) Mean ± SEM responses to light and dark, L- and S-opsin-directed squares (upper and lower panels, respectively) appearing in the RF center for neurons in (D)–(F).

(legend continued on next page)

reflecting center- versus surround-based mechanisms (Figure 4C). Since only the latter has been implicated in color-opponent responses of mRGCs,[10,12,25] in some experiments (n = 15) we tested for the presence of melanopsin input by applying full field Mel. High and low light stimuli (Figure S4J), similar to those employed above. As expected, these stimuli identified a subset of MR cells (n = 20/68; Figures S4K and S4L). This group contained a similar proportion of opponent neurons to the non-MR cells (Figure S4M; n = 5/20 versus n = 14/48) and both groups included cells with opponent RFs or full-field opponency. Insofar as we encountered several examples of apparent center opponency among the (modest) sample of MR-opponent cells tested (n = 2 L-ON/S-OFF and 1 S-ON/L-OFF), input from M5 or M4 mRGCs is unlikely to fully account for the opponent properties of these LGN cells.

Collectively, our data provide new insight into chromatic processing in the mouse visual system. Color opponency is specifically enriched within a chromatic zone encompassing medial portions of the LGN where, consistent with known LGN visuotopic organization[22,34] and behavioral evidence for robust wavelength discrimination in central-upper visual field,[9] color-opponent cells have RFs located between ~10° below to 68° above the mouse head (corresponding to between ~32° dorsal and 46° ventral of the retinal midline[15,36]). Previous studies have provided evidence for mechanisms that could support chromatic discrimination in the corresponding portions of the retina, albeit with divergent estimates as to the prevalence (2%–30%) of RGC color opponency.[7,10,12–14] Our finding that >25% of mouse LGN neurons exhibit color opponency is greater than found in previous LGN recordings (~10%) that did not use cone-selective stimuli[15] but broadly aligns with upper estimates from RGC recordings.[13] Significantly, however, the properties observed for LGN opponent responses diverge in a number of ways from proposed retinal mechanisms. Indeed, whereas rods have been suggested to provide opponent responses in ventral retina (where "pure" M-cones are scarce[13]), we find that cones, rather than rods, account for the color-opponent responses observed in LGN neurons across a range of light intensities typical of twilight-daylight transitions.[33] We further find that LGN chromatic responses are not overtly dependent on the retinal cone opsin expression gradient, as reported for alpha-RGCs,[10] nor do they seem solely reliant on the surround-based mechanisms previously identified in other RGC types.[11–13] Our modeling suggests cone-subtype-specific retinal circuits[7,8,35] are likely important for aspects of the responses. However, the opponency observed for small spatially localized stimuli in almost half of color-opponent LGN neurons (comparatively rare in RGCs[13]) implies also that central mechanisms (e.g., convergent input from multiple RGC types,[37–39] local interneuron, or even cortical/collicular feedback) may play a role in generating the chromatic properties of these thalamic neurons.

While our data establish cone photoreception as the dominant source of color opponency in the LGN across the upper mesopic to photopic range, we also do not exclude the possibility that other photoreceptive signals could influence color vision. Mechanisms supporting opponency have been identified for mRGC subtypes known to project to the LGN[10,12,40] and rod-cone opponency has been reported for another mouse RGC type.[11] We did not find any direct evidence for meaningful contributions of either rods or melanopsin to opponent responses under the conditions studied here. It is possible, however, that such actions might become apparent under lower light intensities or following more extensive adaption (rods) or for lower spatiotemporal frequency stimuli (melanopsin), and thereby extend the operating range of mouse color vision. Nonetheless, we show here that cones alone support a diverse capacity for color discrimination that operates for small spatially localized stimuli and diffuse changes in illumination across a range of light levels, providing a robust substrate for mouse color vision.

STAR★METHODS

Detailed methods are provided in the online version of this paper and include the following:

- KEY RESOURCES TABLE
- RESOURCE AVAILIABILITY
  - Lead contact
  - Materials availability
  - Data and code availability
- EXPERIMENTAL MODEL AND SUBJECT DETAILS
  - Mice
- METHOD DETAILS
  - *In vivo* Electrophysiology
  - Full field visual stimuli
  - Spatially patterned stimuli
  - Histology & Immunohistochemistry
- QUANTIFICATION AND STATISTICAL ANALYSIS

SUPPLEMENTAL INFORMATION

Supplemental information can be found online at https://doi.org/10.1016/j.cub.2021.05.024.

ACKNOWLEDGMENTS

This work was supported by grants from the Biotechnological and Biological Sciences Research Council UK (B/N014901/1) and Wellcome Trust (210684/Z/18/Z). The authors thank Franck Martial for assistance in constructing and programming the spatially patterned visual stimulator.

AUTHOR CONTRIBUTIONS

T.M.B., R.J.L., and J.W.M. designed the experiments. J.W.M., A.P., A.J.W., and C.W. performed the experiments. J.W.M., A.P., and T.M.B. performed the analysis. T.M.B. and R.J.L. wrote the manuscript, with assistance from other authors.

DECLARATION OF INTERESTS

The authors declare no competing interests.

(J–L) Mean ± SEM responses to full-field 0.25 Hz L- and S-cone opsin-directed modulations for neurons in (D)–(I).
See also Figure S4 for additional data on RF properties of opponent and non-opponent cells contributing to (B)–(L) and responses to additional stimuli used for cell classification.

CellPress
OPEN ACCESS

Received: December 15, 2020
Revised: April 23, 2021
Accepted: May 13, 2021
Published: June 9, 2021

REFERENCES

1. Price, T.D. (2017). Sensory drive, color, and color vision. Am. Nat. *190*, 157–170.
2. Spitschan, M., Lucas, R.J., and Brown, T.M. (2017). Chromatic clocks: color opponency in non-image-forming visual function. Neurosci. Biobehav. Rev. *78*, 24–33.
3. Baden, T., and Osorio, D. (2019). The retinal basis of vertebrate color vision. Annu. Rev. Vis. Sci. *5*, 177–200.
4. Thoreson, W.B., and Dacey, D.M. (2019). Diverse cell types, circuits, and mechanisms for color vision in the vertebrate retina. Physiol. Rev. *99*, 1527–1573.
5. Applebury, M.L., Antoch, M.P., Baxter, L.C., Chun, L.L., Falk, J.D., Farhangfar, F., Kage, K., Krzystolik, M.G., Lyass, L.A., and Robbins, J.T. (2000). The murine cone photoreceptor: a single cone type expresses both S and M opsins with retinal spatial patterning. Neuron *27*, 513–523.
6. Baden, T., Schubert, T., Chang, L., Wei, T., Zaichuk, M., Wissinger, B., and Euler, T. (2013). A tale of two retinal domains: near-optimal sampling of achromatic contrasts in natural scenes through asymmetric photoreceptor distribution. Neuron *80*, 1206–1217.
7. Nadal-Nicolás, F.M., Kunze, V.P., Ball, J.M., Peng, B.T., Krishnan, A., Zhou, G., Dong, L., and Li, W. (2020). True S-cones are concentrated in the ventral mouse retina and wired for color detection in the upper visual field. eLife *9*, e56840.
8. Haverkamp, S., Wässle, H., Duebel, J., Kuner, T., Augustine, G.J., Feng, G., and Euler, T. (2005). The primordial, blue-cone color system of the mouse retina. J. Neurosci. *25*, 5438–5445.
9. Denman, D.J., Luviano, J.A., Ollerenshaw, D.R., Cross, S., Williams, D., Buice, M.A., Olsen, S.R., and Reid, R.C. (2018). Mouse color and wavelength-specific luminance contrast sensitivity are non-uniform across visual space. eLife *7*, e31209.
10. Chang, L., Breuninger, T., and Euler, T. (2013). Chromatic coding from cone-type unselective circuits in the mouse retina. Neuron *77*, 559–571.
11. Joesch, M., and Meister, M. (2016). A neuronal circuit for colour vision based on rod-cone opponency. Nature *532*, 236–239.
12. Stabio, M.E., Sabbah, S., Quattrochi, L.E., Ilardi, M.C., Fogerson, P.M., Leyrer, M.L., Kim, M.T., Kim, I., Schiel, M., Renna, J.M., et al. (2018). The M5 cell: a color-opponent intrinsically photosensitive retinal ganglion cell. Neuron *97*, 150–163.e4.
13. Szatko, K.P., Korympidou, M.M., Ran, Y., Berens, P., Dalkara, D., Schubert, T., Euler, T., and Franke, K. (2020). Neural circuits in the mouse retina support color vision in the upper visual field. Nat. Commun. *11*, 3481.
14. Ekesten, B., and Gouras, P. (2005). Cone and rod inputs to murine retinal ganglion cells: evidence of cone opsin specific channels. Vis. Neurosci. *22*, 893–903.
15. Denman, D.J., Siegle, J.H., Koch, C., Reid, R.C., and Blanche, T.J. (2017). Spatial organization of chromatic pathways in the mouse dorsal lateral geniculate nucleus. J. Neurosci. *37*, 1102–1116.
16. Ekesten, B., and Gouras, P. (2008). Cone inputs to murine striate cortex. BMC Neurosci. *9*, 113.
17. Govardovskii, V.I., Fyhrquist, N., Reuter, T., Kuzmin, D.G., and Donner, K. (2000). In search of the visual pigment template. Vis. Neurosci. *17*, 509–528.
18. Jacobs, G.H., and Williams, G.A. (2007). Contributions of the mouse UV photopigment to the ERG and to vision. Doc. Ophthalmol. *115*, 137–144.
19. Tikidji-Hamburyan, A., Reinhard, K., Storchi, R., Dietter, J., Seitter, H., Davis, K.E., Idrees, S., Mutter, M., Walmsley, L., Bedford, R.A., et al. (2017). Rods progressively escape saturation to drive visual responses in daylight conditions. Nat. Commun. *8*, 1813.
20. Smallwood, P.M., Olveczky, B.P., Williams, G.L., Jacobs, G.H., Reese, B.E., Meister, M., and Nathans, J. (2003). Genetically engineered mice with an additional class of cone photoreceptors: implications for the evolution of color vision. Proc. Natl. Acad. Sci. USA *100*, 11706–11711.
21. Hayter, E.A., and Brown, T.M. (2018). Additive contributions of melanopsin and both cone types provide broadband sensitivity to mouse pupil control. BMC Biol. *16*, 83.
22. Piscopo, D.M., El-Danaf, R.N., Huberman, A.D., and Niell, C.M. (2013). Diverse visual features encoded in mouse lateral geniculate nucleus. J. Neurosci. *33*, 4642–4656.
23. Ecker, J.L., Dumitrescu, O.N., Wong, K.Y., Alam, N.M., Chen, S.K., LeGates, T., Renna, J.M., Prusky, G.T., Berson, D.M., and Hattar, S. (2010). Melanopsin-expressing retinal ganglion-cell photoreceptors: cellular diversity and role in pattern vision. Neuron *67*, 49–60.
24. Brown, T.M., Gias, C., Hatori, M., Keding, S.R., Semo, M., Coffey, P.J., Gigg, J., Piggins, H.D., Panda, S., and Lucas, R.J. (2010). Melanopsin contributions to irradiance coding in the thalamo-cortical visual system. PLoS Biol. *8*, e1000558.
25. Schmidt, T.M., Alam, N.M., Chen, S., Kofuji, P., Li, W., Prusky, G.T., and Hattar, S. (2014). A role for melanopsin in alpha retinal ganglion cells and contrast detection. Neuron *82*, 781–788.
26. Do, M.T.H. (2019). Melanopsin and the intrinsically photosensitive retinal ganglion cells: biophysics to behavior. Neuron *104*, 205–226.
27. Brown, T.M., Allen, A.E., al-Enezi, J., Wynne, J., Schlangen, L., Hommes, V., and Lucas, R.J. (2013). The melanopic sensitivity function accounts for melanopsin-driven responses in mice under diverse lighting conditions. PLoS ONE *8*, e53583.
28. Brown, T.M., Tsujimura, S., Allen, A.E., Wynne, J., Bedford, R., Vickery, G., Vugler, A., and Lucas, R.J. (2012). Melanopsin-based brightness discrimination in mice and humans. Curr. Biol. *22*, 1134–1141.
29. Allen, A.E., Storchi, R., Martial, F.P., Bedford, R.A., and Lucas, R.J. (2017). Melanopsin contributions to the representation of images in the early visual system. Curr. Biol. *27*, 1623–1632.e4.
30. Rivlin-Etzion, M., Zhou, K., Wei, W., Elstrott, J., Nguyen, P.L., Barres, B.A., Huberman, A.D., and Feller, M.B. (2011). Transgenic mice reveal unexpected diversity of on-off direction-selective retinal ganglion cell subtypes and brain structures involved in motion processing. J. Neurosci. *31*, 8760–8769.
31. Marshel, J.H., Kaye, A.P., Nauhaus, I., and Callaway, E.M. (2012). Anterior-posterior direction opponency in the superficial mouse lateral geniculate nucleus. Neuron *76*, 713–720.
32. Brown, T.M., Allen, A.E., Wynne, J., Paul, D.L., Piggins, H.D., and Lucas, R.J. (2011). Visual responses in the lateral geniculate evoked by Cx36-independent rod pathways. Vision Res. *51*, 280–287.
33. Walmsley, L., Hanna, L., Mouland, J., Martial, F., West, A., Smedley, A.R., Bechtold, D.A., Webb, A.R., Lucas, R.J., and Brown, T.M. (2015). Colour as a signal for entraining the mammalian circadian clock. PLoS Biol. *13*, e1002127.
34. Howarth, M., Walmsley, L., and Brown, T.M. (2014). Binocular integration in the mouse lateral geniculate nuclei. Curr. Biol. *24*, 1241–1247.
35. Breuninger, T., Puller, C., Haverkamp, S., and Euler, T. (2011). Chromatic bipolar cell pathways in the mouse retina. J. Neurosci. *31*, 6504–6517.
36. Sterratt, D.C., Lyngholm, D., Willshaw, D.J., and Thompson, I.D. (2013). Standard anatomical and visual space for the mouse retina: computational reconstruction and transformation of flattened retinae with the Retistruct package. PLoS Comput. Biol. *9*, e1002921.
37. Rompani, S.B., Müllner, F.E., Wanner, A., Zhang, C., Roth, C.N., Yonehara, K., and Roska, B. (2017). Different modes of visual integration in the lateral geniculate nucleus revealed by single-cell-initiated transsynaptic tracing. Neuron *93*, 1519.

38. Liang, L., Fratzl, A., Goldey, G., Ramesh, R.N., Sugden, A.U., Morgan, J.L., Chen, C., and Andermann, M.L. (2018). A fine-scale functional logic to convergence from retina to thalamus. Cell *173*, 1343–1355.e24.

39. Román Rosón, M., Bauer, Y., Kotkat, A.H., Berens, P., Euler, T., and Busse, L. (2019). Mouse dLGN receives functional input from a diverse population of retinal ganglion cells with limited convergence. Neuron *102*, 462–476.e8.

40. Estevez, M.E., Fogerson, P.M., Ilardi, M.C., Borghuis, B.G., Chan, E., Weng, S., Auferkorte, O.N., Demb, J.B., and Berson, D.M. (2012). Form and function of the M4 cell, an intrinsically photosensitive retinal ganglion cell type contributing to geniculocortical vision. J. Neurosci. *32*, 13608–13620.

41. Brainard, D.H. (1997). The Psychophysics Toolbox. Spat. Vis. *10*, 433–436.

42. Pelli, D.G. (1997). The VideoToolbox software for visual psychophysics: transforming numbers into movies. Spat. Vis. *10*, 437–442.

43. Pachitariu, M., Steinmetz, N., Kadir, S., Carandini, M., and Harris, K. (2016). Kilosort: realtime spike-sorting for extracellular electrophysiology with hundreds of channels. bioRxiv. https://doi.org/10.1101/06148.

44. Allen, A.E., Storchi, R., Martial, F.P., Petersen, R.S., Montemurro, M.A., Brown, T.M., and Lucas, R.J. (2014). Melanopsin-driven light adaptation in mouse vision. Curr. Biol. *24*, 2481–2490.

45. Wu, S.M., Gao, F., and Pang, J.J. (2004). Synaptic circuitry mediating light-evoked signals in dark-adapted mouse retina. Vision Res. *44*, 3277–3288.

46. Nathan, J., Reh, R., Ankoudinova, I., Ankoudinova, G., Chang, B., Heckenlively, J., and Hurley, J.B. (2006). Scotopic and photopic visual thresholds and spatial and temporal discrimination evaluated by behavior of mice in a water maze. Photochem. Photobiol. *82*, 1489–1494.

47. Pienaar, A., Walmsley, L., Hayter, E., Howarth, M., and Brown, T.M. (2018). Commissural communication allows mouse intergeniculate leaflet and ventral lateral geniculate neurons to encode interocular differences in irradiance. J. Physiol. *596*, 5461–5481.

48. Paxinos, G.F., and Franklin, K.G.J. (2001). The Mouse Brain in Stereotaxic Coordinates, Second Edition (Academic Press).

CellPress
OPEN ACCESS

STAR★METHODS

KEY RESOURCES TABLE

REAGENT or RESOURCE	SOURCE	IDENTIFIER
Antibodies		
MWS/LWS opsin, rabbit polycolonal antibody	Sigma Aldritch	Cat# AB5405; RRID: AB_177456
SWS opsin, rabbit polycolonal antibody	Sigma Aldritch	Cat# AB5407; RRID: AB_177457
Goat anti-Rabbit IgG secondary antibody, Alexa Fluor 488	Invitrogen	Cat# A-11008; RRID: AB_143165
Donkey anti-Rabbit IgG secondary antibody, Alexa Fluor 555	Invitrogen	Cat# A-31572; RRID: AB_162543
Chemicals, peptides, and recombinant proteins		
CM-DiI	Fisher Scientific	Cat# V22888
Experimental models: Organisms/strains		
Mouse: Opn1mwR	Prof. Jeremy Nathans, Johns Hopkins University	MGI Cat# 2678771; RRID: MGI:2678771
Software and algorithms		
MATLAB R2018a	MathWorks	https://uk.mathworks.com/products/matlab.html
SPSS version 25	IBM	https://www.ibm.com/uk-en/analytics/spss-statistics-software
Psychophysics toolbox	Prof. David Brainard;[41] Prof. Denis Pelli[42]	http://psychtoolbox.org/
LabView 8.6	National Instruments	http://www.ni.com/labview/
Kilosort	Dr. Marius Pachitariu[43]	https://github.com/cortex-lab/KiloSort
OfflineSorter version 3	Plexon	http://www.plexon.com/products/offline-sorter
Other		
4X8 multielectode recording array	Neuronexus	Cat# A4x8-5mm-200-50-177
4x2 tetrode recording array	Neuronexus	Cat# A4x2-tet-150-200-121
4x16 polytrode recording array	Neuronexus	Cat# A4x16-Poly2-5mm-23 s-200-177
4x64 polytrode recording array	Neuronexus	Cat# A4x64-Poly2-5mm-23 s-200-177
405nm LED	Thorlabs	Cat# M405L4
460nm LED	Thorlabs	Cat# M455L4
620nm LED	Thorlabs	Cat# M617L4
Unmounted Reflective Ø25 mm ND Filter, Optical Density: 1.0	Thorlabs	Cat# ND10B
1/4” x 36,” Flexible Fiber Optic Light Guide	Edmund Optics	Cat# NT42-347
Custom spatial projector system	Rob Lucas[29,44]	N/A

RESOURCE AVAILIABILITY

Lead contact

Further information and requests for resources and reagents should be directed to and will be fulfilled by the Lead Contact, Tim Brown (Timothy.Brown@manchester.ac.uk)

Materials availability

This study did not generate new unique reagents.

Data and code availability

Raw data and analysis code will be provided upon request by the Lead Contact Tim Brown (timothy.brown@manchester.ac.uk).

EXPERIMENTAL MODEL AND SUBJECT DETAILS

Mice

All experiments were performed in accordance with the Animals (Scientific Procedures) Act of 1986 (United Kingdom) and received institutional and UK Home Office approval. Mice were bred and housed at the University of Manchester in a 12:12 h light dark cycle at 22°C with food and water available *ad libitum*. All experiments were performed in adult male *Opn1mw*^{R} mice (80-160 days old).

METHOD DETAILS

In vivo Electrophysiology

Mice were anesthetized with urethane (1.55 g/kg i.p; Sigma-Aldrich, Dorset, UK) and prepared for stereotaxic surgery as described previously.[34] In brief, a craniotomy (< 1mm diameter) was placed 2.3mm lateral and 2.5mm posterior to bregma, atropine (1% in saline; Sigma-Aldrich) was applied to the eyes to dilate the pupils and a drop of mineral oil (Sigma-Aldrich) applied subsequently to retain corneal moisture. Recordings employed silicon-substrate multielectrode arrays (NeuroNexus, MI, USA) with 4 shanks (spaced 200μm, shank width = 15μm) bearing a total of 32 (A4x8-5mm-200-50-177; n = 18 mice; A4x2-tet-150-200-121; n = 5 mice), 64 (A4x16-Poly2-5mm-23 s-200-177; n = 23 mice) or 256 recording sites (A4x64-Poly2-5mm-23 s-200-177; n = 4 mice). Immediately prior to use, recording probes were coated in CM-DiI (V22888; Fisher Scientific, Loughborough, UK) to allow post hoc visualization in histological images, before being inserted into the brain to target the LGN complex. Prior to neurophysiological recording, mice were left for 30min to dark adapt and allow neural activity to stabilize. In a few cases where we used 32channel recording probes (n = 5 mice), after completion of the experimental protocol the recording electrode was slowly raised 400μm and the protocol repeated to collect a second set of data (from a non-overlapping portion of the LGN complex).

For 32 and 64 channel recordings, wideband neuronal data was acquired using a Recorder64 data acquisition system (Plexon, TX, USA), amplified for a gain of 3500X, digitized at 40kHz and stored continuously in a 16bit format. For 256 channel recordings data was acquired using a Smartbox system (NeuroNexus) sampling at 20 kHz. All subsequent processing was identical across experiments and we did not detect any quantitative or qualitative differences between single units isolated from data generated using the two systems. Single unit activity was isolated using an automated template-matching based algorithm (Kilosort[43]). Identified clusters and unassigned multiunit spikes were then exported to Offline Sorter (Plexon), as 'virtual tetrodes' (spike waveforms detected across 4 adjacent channels) for manual refinement.[34] Single unit isolation was confirmed by reference to MANOVA F statistics, J3 and Davies-Bouldin validity metrics and the presence of a distinct refractory period (> 1.5ms) in the interspike interval distribution.

Full field visual stimuli

Light measurements were performed using a calibrated spectroradiometer (Bentham instruments, Reading, UK). Subsequent quantification as effective photon flux for each class of retinal opsin present in *Opn1mw*^{R} was then performed by reference to the known opsin sensitivities after correction for prereceptoral filtering[17,18] as described previously.[21]

Visual stimuli were generated via a custom light source (components from Thorlabs: NJ, USA and Edmund Optics; York, UK) which combined signals from three LEDs (λmax 405nm, 460nm and 620nm) via dichroic mirrors. Light was delivered via a 7mm diameter flexible fiber optic light guide positioned 5mm from the mouse's contralateral eye and enclosed within an internally reflective plastic cone to provide approximately full field illumination. An equivalent assembly was positioned over the ipsilateral eye, providing light (where required) from a single 405nm LED. LED intensity was controlled dynamically via a PC running LabVIEW and a USB-6343 DAQ board (National Instruments, TX, USA) and via neutral density (ND) filter wheels, allowing for spectrally neutral 10 to 10,000-fold (ND1-ND4) reductions in light intensity.

The visual stimuli employed have been described and validated in detail, previously.[21] To identify cell receiving input from mRGCs ('MR cells') we compared responses to i) 460nm light step ('Mel. High') and ii) a combination of 405nm and 620nm light ('Mel. Low') providing equivalent photon flux for L- and S-cone opsins but providing ~500-fold lower photon flux for melanopsin and rods (Figure S1B). Stimuli were presented in interleaved fashion as 10 s steps from a background of darkness (60 s ISI; 10 repeats/stimulus at ND2-0). Across this intensity range all stimuli are expected to be sufficiently high to induce transient rod saturation (11.8 Log rod-effective photons/cm^2/s for Mel. Low at ND2).[32,45,46] For selective manipulation of cone photoreception, we calibrated the three-primary system to re-create the pattern of photoreceptor activation occurring for a wildtype mouse experiencing natural daylight (Figure S1C). We then adjusted the spectra (via independently modulating brightness of each LED) so as to change activation of L- and/or S- cone opsins, in isolation, unison or antiphase, by up to ± 75% relative to the background (equivalent to a 0.85 log unit or 7-fold change in apparent brightness for the stimulated opsin; Figure S1D). Stimuli were applied as 0.25Hz square-wave modulations providing 15%–75% Michelson contrast (with a smooth 40ms transition between 'bright' and 'dim' phases). The stimulus set was presented as interleaved blocks of 6 cycles of L_{Only}, S_{Only}, L+S and L-S isolating stimuli running from low to high contrast. The full protocol was then repeated 5 times to provide 30 repeats for each stimulus. For the highest contrast L- and S-cone isolating stimuli, effective change in brightness for the silenced cone subtypes were < 0.2% Michelson and effective changes in rod and melanopsin activation < 6% for all stimuli. As part of the protocol above, we also presented a stimulus designed to provide 45% rod contrast (and 43.2% melanopsin contrast) while providing negligible contrast for cones (< 0.05%; Figure S2B).

In some experiments, prior to undertaking the protocol above, we first applied interleaved cycles of the 75% contrast cone isolating stimuli at a range of logarithmically spaced light intensities (ND4-ND0; 5xblocks of 6 cycles/stimulus as above). To probe for cone-independent influences on neural activity, this stimulus set also included spectrally neutral; 75% contrast modulations in light intensity. Across all experiments employing cone isolating stimuli, background light intensity at the eye ipsilateral to our recording sites was set to approximate that for the stimulated eye via the 405nm LED system (which provides near equal activation of all mouse opsin classes[34]).

In other experiments, we probed rod and cone contributions across a range of background light intensities using a 10Hz white noise stimulus where S- and L-cone and rhodopsin contrast was independently modulated by ± 65% in a pseudorandom sequence (10min duration at each intensity). To achieve this degree of independent rod and cone contrast, these experiments employed a different background stimulus to that used for other studies which had reduced overall irradiance for S-cone opsin (Figure 2G).

Spatially patterned stimuli

Initial mapping of RF positions was performed in 26/27 of the experiments used for assessment of cone responses to full field stimuli and was performed similarly to that described in Pienaar et al.[47] Stimuli were delivered via an LCD display (width: 26.8cm height: 47.4cm; Hanns-G HE225DPB) angled at 45° from vertical and placed at a distance of ~21cm from the contralateral eye to occupying ~96° of elevation. On the azimuthal plane the display subtended ~63° and was placed sequentially at 2-3 adjacent positions (starting directly in front of the mouse) to cover the majority of the nasal-temporal visual axis. Across individual experiments, test stimuli were therefore presented across ~33% (two positions) to 57% (3 monitor positions) of the full visual field (assumed to encompass 180° in azimuth and elevation). In the manuscript, elevation coordinates are specified relative to mouse snout axis. Based on the tilt of the mouse eye (~22°), the display covered substantial portions of the central and ventral retina (between ~30° below to ~70° above the retinal midpoint depending on precise monitor positions across experiments). The ipsilateral eye received 410nm LED light as above at an equivalent irradiance to the background irradiance in the experimental room (~14 log photons/cm2/s). Stimuli were generated and controlled via MATLAB (Mathworks, MA, USA) using the Psychophysics toolbox[41,42] and comprised light or dark bars (430 and 3.3 scotopic cd/m2 respectively, occupying ~7° visual angle) superimposed on a background of the opposite polarity. Vertical or horizontal bars appeared at random locations (~1.5° increments covering the display) for 250ms followed by a blank screen for 250ms (8 repeats per orientation/polarity/screen location).

For RF mapping using cone-opsin isolating stimuli, we used a custom projector system, as previously described.[29,44] Briefly, the output of 3 LEDs (λmax 405, 455, 630 nm; Phlatlight PT-120 Series, Luminus Devices, CA, USA) were modulated via an UNO32 microcontroller (Digilent, WA, USA) to generate the desired spectra. Light from the LEDs was directed onto a digital mirror device projector (DLP LightCommander; LogicPD, MN, USA). Images were back projected onto a screen, covering 30°x40° visual angle and positioned to target the central part of the dorsal-ventral visual axis. As above, the screen was positioned sequentially at three adjacent locations on the nasal-temporal axis to expand coverage of the central visual field. Here stimuli were presented as ~6°x6°, 72% Michelson contrast, squares appearing at random locations (from an 11x14 grid) on the screen for 250ms, followed by 250ms blank screen, so as to provide ~0.8 log unit increments of decrements in light intensity selectively for L- or S-cone opsin (Figure 4A). To prevent off-target effects on cells with RFs located at the edge of the visual display, the screen was surround by an annulus comprising RGB and violet LEDS (λmax = 630, 517, 460 and 400nm) covered by an opal polypropylene diffuser, with LED intensity set to match the display background. For these experiments, we also evaluated responses to wide-field L- and S-cone opsin isolating modulations (0.25 Hz square wave; 15 repeats). These stimuli were either delivered as full-screen flashes (n = 8 experiments) or delivered via a fiber optic placed close to the eye to cover the full visual field (n = 15 experiments). For the latter, stimuli were generated via multispectral light device (SPECTRA X light engine; Lumencor, OR, USA). This device contains 6 light sources of which 3 (λmax 395nm, 435nm, 635nm) were used to generate the cone-isolating stimuli comparable to those used for RF mapping. There was no discernible difference between responses observed for wide-field cone-isolating stimuli applied via this two systems. For experiments using the Lumencor system, we also evaluated responses to full field L- and S-cone opsin matched Mel. High and Low stimuli (1-log unit difference in melanopsin excitation), produced using divergent combinations of 395, 435, 485, 510 and 580nm sources (Figure S4J).

Histology & Immunohistochemistry

After each experiment, brains were removed and placed into 4% paraformaldehyde for 48 h before overnight cryoprotection in 30% sucrose. Brains were then frozen with dry ice and sectioned coronally (width = 100μm) using a freezing sledge microtome before mounting with Vectashield (Vector laboratories, UK) to glass slides and coverslipping. Sections were imaged under an upright light microscope (BX51; Olympus, UK) with appropriate filter sets for visualization of DiI fluorescence and images acquired with a Coolsnap HQ camera (Photometrics, USA). Resulting images were scaled and aligned with best matching coronal panels from the mouse atlas[48] with the anatomical location for each cell estimated based on the known geometry of the recording array and the corresponding recording site location were largest spike amplitudes were detected. For display, estimated unit locations were mapped onto a single mid-coronal anatomical template of the LGN.

For retinal immunohistochemistry mice were anesthetized with urethane and perfused with 4% paraformaldehyde (methanol free). Eyes were then stored in methanol-free 4% paraformaldehyde prior to further processing. Retinas were dissected from fixed eyes and immunohistochemistry performed on free-floating retinas. Retinas were washed with 1% PBS-T, blocked with 10% mixed goat and donkey serum in PBS for 3 h, then labeled using polyclonal antibodies against MWS/LWS opsin (AB5405, Sigma Aldritch;

1:250 dilution) and left overnight at room temperature. The following day retinas were washed with 0.2% PBS-T and then incubated with fluorescent secondary antibodies for 3 h (A31572, Invitrogen; 1:500 dilution). Retinas were washed again in 0.2% PBS-T and labeled against SWS opsin (AB5407, Sigma Aldritch; 1:500 dilution) and left overnight at room temperature. On the last day retinas were washed in 0.2% PBS-T and then incubated with fluorescent secondary antibody (A11008, Invitrogen; 1:500) for 3 h before being washed in PBS and mounted. Primary antibodies were added in a mixture of 0.2% PBS-T with 2.5% mixed goat and donkey serum. Secondary antibodies were added in a mixture of 0.2% PBS-T with 5% mixed goat and donkey serum. Sections were imaged with an Axio Imager.D2 upright microscope and captured using a Coolsnap Hq2 camera (Photometrics) through Micromanager software v1.4.23.

QUANTIFICATION AND STATISTICAL ANALYSIS

For neuronal classification, cells were considered melanopsin responsive when i) steady state firing during the last 5 s of Mel. High stimulus was significantly greater than that observed in response to Mel. Low (at any of the tested intensities) while firing rate during initial components of the response (first 250ms) was not significantly different (unpaired t test, $p > 0.05$). For quantification we extracted the mean change in firing rate for each cell (relative to the preceding 10 s baseline) across early (first 250ms) or late (last 5 s) elements of the response and analyzed with a mixed effects linear model (SPSS v. 25, IBM, NY, USA). For this analysis, the mouse from which the cell was recorded was included as a random factor and intensity and stimulus type as fixed, repeated–measures, factors

For analysis of neuronal responses to full field square wave cone-isolating stimuli, spike counts were binned (100 bins/stimulus cycle; smoothed with a 5-bin boxcar filter) and peak-trough amplitudes extracted. To remove the effect of random variations in baseline firing, we calculated equivalent estimates based on shuffled data (spike counts shuffled in time independently for each trial). Cells were considered responsive when the measured response amplitude exceed the 95% confidence limits of responses assessed from shuffled data (100 repeats), with the mean shuffled response subsequently subtracted from the true response. Response polarity (ON versus OFF) was assessed based on the stimulus phase where we observed the largest absolute deviation in spike rates from the mean and the sign (positive versus negative) of that response. Cells were designated as color opponent where we observed significant responses (as defined above) of opposite sign to L_{Only} and S_{Only} contrast or in cases where responses to one of the two opsins was not detectable but the mean response to the L-S stimuli (analyzed above) was significantly greater than that to the L+S stimulus (t test, $p < 0.05$). For comparisons of contrast response functions we used a modified $\chi 2$-peridogram[21] to quantify the percentage variance within the timeseries that was accounted for by a rhythmic process with the same periodicity as the stimulus. For other analysis we compared (signed) peak-trough response amplitude. For analysis of white noise data we calculated spike triggered averages (STAs) of rod, L- and S-cone contrasts and extracted peak-trough amplitudes (with response polarity determined based on whether increased or decreased contrast immediately proceeded spike occurrence). For all relevant group comparisons of responses between stimuli described above we employed mixed effects linear model with the mouse in which each cell was recorded as a random factor and contrast/intensity and stimulus type as fixed, repeated-measures, factors as appropriate.

For analysis of RF parameters we first calculated the response to each stimulus as a function of where it appeared on the screen (50ms epoch of peak firing occurring within 35-125ms after bar appearance, relative to mean firing during preceding 100ms). Data for light increments and decrements were combined for each location by subtracting responses to light versus dark bars/squares (for ON or OFF responses) or adding these (ON-OFF cells) as appropriate. The resulting response measures were then fit with 1D (bar stimuli) or 2D Gaussian (cone-isolating squares) to estimate the RF center position (MATLAB).

For analysis of cell densities and response preferences as a function of LGN location, cells were binned based on estimated anatomical location (as described above) using a moving circular window of radius = 150μm. Within each bin we then calculated the percentage of cells of the relevant type or the average of responses preference, as appropriate. The overall size of populations used for binning as a function of anatomical location are shown in Figure S1O (minimum 10 cells/bin).

For retinal 'random-wiring' modeling, we simulated strips of the dorsal-ventral retina (100 random strips per model) based on the published distributions of S-only, M-only and M-/S- opsin co-expressing cones (data from Figure 2D in Baden et al.[6] extracted using WebPlotDigitizer 4.2). For modeling reported in Figure S2 of this manuscript we allowed the relative expression of S-opsin versus total cone opsin expression to vary between 10%–90% in line with the overall dorsal-ventral gradient in S-cone expression, however other models where expression was fixed at 50% (or varied stochastically between 10 and 90%) produced near identical results. We then simulated the effective S- and M-/L-cone contrast expected for our experimental stimuli experienced by center surround neurons with a range of RF diameters between 6 and 20° (the typical range for LGN neurons[15,22,34]) varying weighting of center surround components between 0.5 and 1 and varying degrees of sampling from the cone population within the RF (between 1 and 100% of the cones in that region, randomly determined). Surround size was fixed throughout at 3xRF center diameter.

Current Biology, Volume *31*

Supplemental Information

Extensive cone-dependent spectral opponency within a discrete zone of the lateral geniculate nucleus supporting mouse color vision

Josh W. Mouland, Abigail Pienaar, Christopher Williams, Alex J. Watson, Robert J. Lucas, and Timothy M. Brown

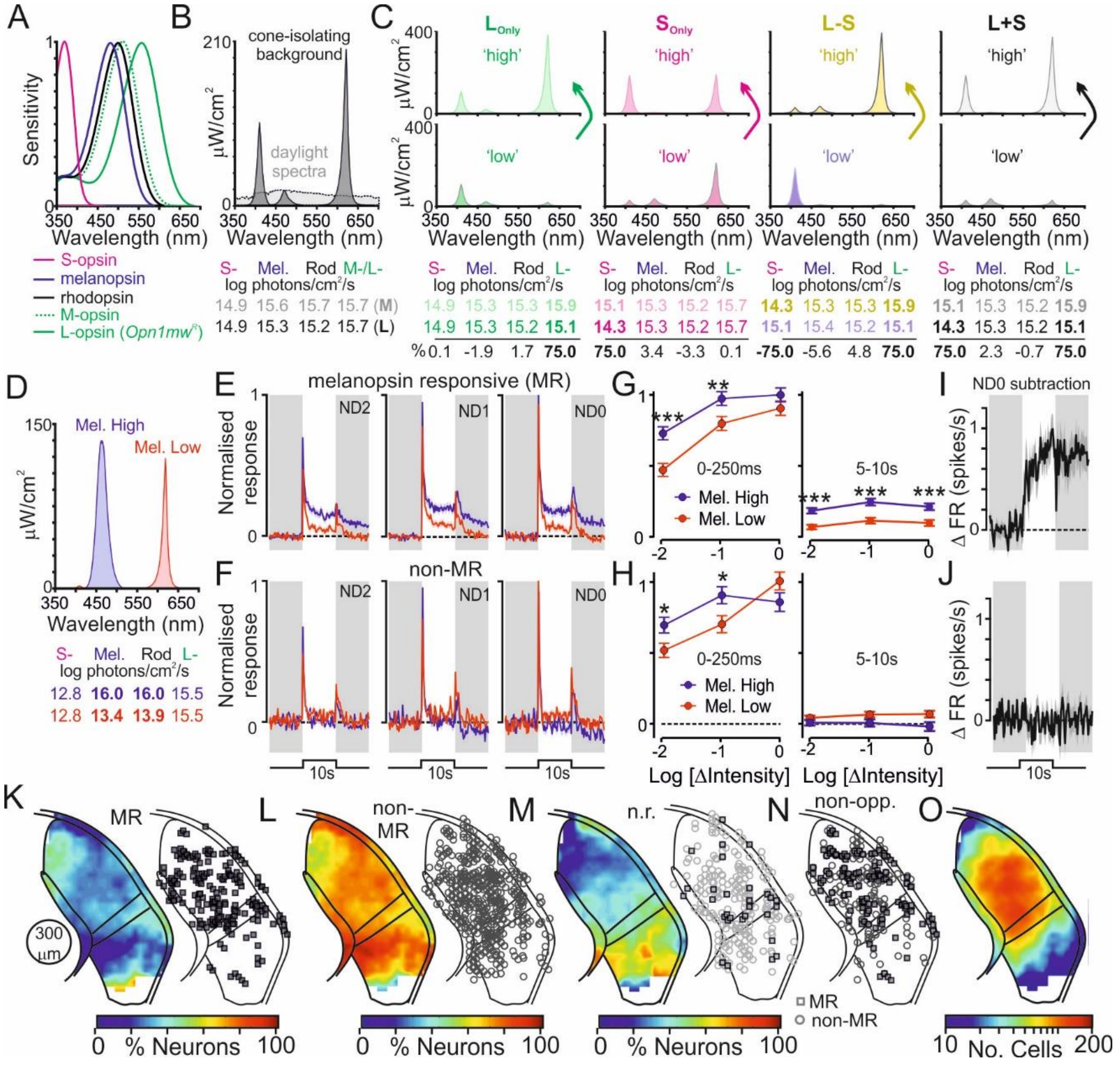

Figure S1. Stimuli used for selective modulation of cone responses and identification of melanopsin responsive neurons. Related to Figure 1. (**A**) Spectral sensitivity of mouse cone opsins, including the long-wavelength shifted opsin in *Opn1mw^R* mice. (**B**) Spectra and quantification of the daylight background used for *Opn1mw^R* LGN recordings and comparison with a natural daylight spectra (quantified according to cone sensitivity in wildtype mice). (**C**) Spectra and quantification for stimulus pairs designed to provide selective contrast (75% Michelson) for L- and/or S-cone opsin, transitions between spectra in the lower and upper panels respectively constitute (left to right): 'L_{Only}', 'S_{Only}', 'L+S-' and 'L+S+' stimuli. (**D**) Spectra of cone-matched, melanopsin high and low stimuli, with quantification for effective irradiance as above. (**E,F**) Mean±SEM normalised change in firing evoked by Mel. High and Low stimuli presented at 0.01, 0.1 or 1x intensity shown in **D** (ND2-0), for cells categorised as melanopsin responsive (MR; **E**, n=195) or non-MR (**F**, n=494). (**G**,**H**) Mean±SEM normalised response evoked by Mel. High and Low stimuli for MR (**G**) and non-MR (**H**) cells, as a function of light intensity during the first 250ms (left) or last 5s (right) of the light step. Data analysed by mixed-effects linear model with Sidak's post-tests ($\mathbf{G}_{Left}$: Stim.-$F_{1,917}$=20.47, P<0.001, Int.-$F_{2,780}$=34.1, P<0.001, Stim.XInt.- $F_{2,780}$=1.8, P=0.16; $\mathbf{G}_{Right}$: Stim.-$F_{1,1072}$=51.9, P<0.001, Int.- $F_{2,797}$=3.4, P=0.03, Stim.XInt.- $F_{2,797}$=0.19, P=0.83; $\mathbf{H}_{Left}$: Stim.-$F_{1,2873}$=2.8, P=0.1, Int.-$F_{2,2021}$=16.9, P<0.001, Stim.XInt.- $F_{2,2021}$=5.48, P=0.004; $\mathbf{H}_{Right}$: Stim.-$F_{1,2457}$=9.8, P=0.002, Int.-$F_{2,2071}$=0.13, P=0.88, Stim.XInt.- $F_{2,2071}$=0.82,P=0.44). (**I,J**) Mean±SEM difference in firing to Mel. High minus Mel. Low at ND0 for MR (**I**) and non-MR (**J**) cells. (**K,L**) Proportions MR (**K**) vs. non-MR (**L**) LGN neurons as a function of anatomical location (left, binned with 150µm radius window) and corresponding unit locations (right). (**M,N**) Anatomical distribution of LGN neurons (MR

and non-MR combined) that lacked response to cone isolating stimuli (**M**; n.r.) or exhibited non-opponent responses (**N**; corresponding heatmap shown in Figure 1J). (**O**) Overall density of cells used for spatial binning in Figure 1 and S1 (note: log scale).

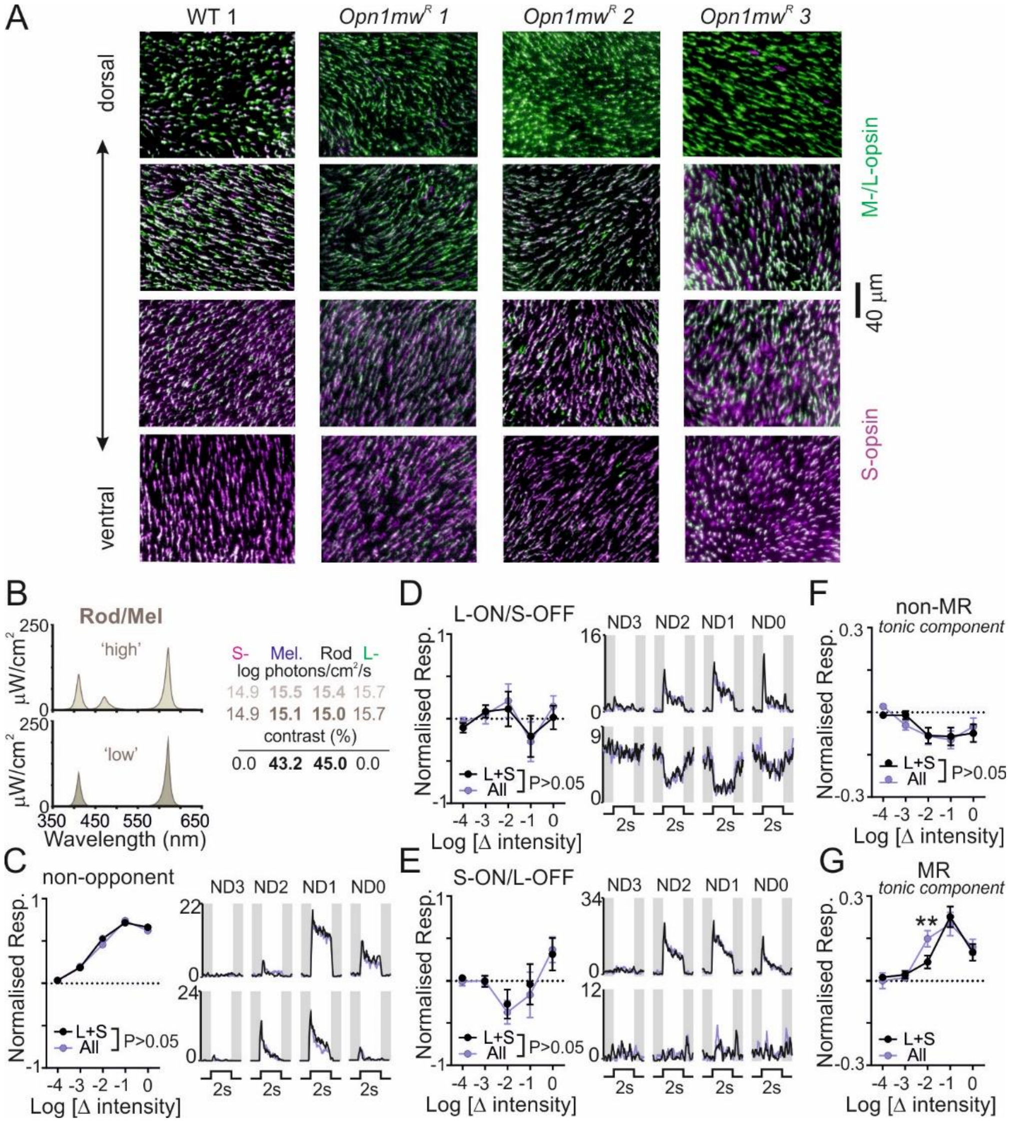

Figure S2. Opsin expression and cone-based response validation in red cone mice. Related to Figure 2. (**A**) Immunohistochemical detection of M-/L- and S-opsin expression across dorsal-ventral portions of retinas from wildtype (WT; left) and *Opn1mw*^{R} mice (mid-right). (**B**) Spectra and quantification for stimulus pairs designed to provide cone-silent contrast for rods and melanopsin (45% rod contrast). (**C-E**) Mean±SEM normalised firing responses (left) and responses of representative neurons (right; in each case upper row shows MR and lower row non-MR neuron) to 75% contrast stimuli modulating both cone types independent of, or concomitant with, changes in rod and melanopsin excitation (right panels; L+S vs. 'All' respectively) at varying background light intensities. Data derives from same neurons in Figure 2D-F (**C**: non-opponent; n=97, responses of n=16 OFF cells sign inverted; **D**: L-ON/S-OFF, n=14; **E**: S-ON/L-OFF, n=13). Data analysed by mixed-effects linear model (**C**: Stim.-$F_{1,884}$=0.6, P=0.45, Int.- $F_{4,408}$=211.3, P<0.001, Stim.XInt.- $F_{4,408}$=0.67, P=0.62; **D**: Stim.-$F_{1,79}$=0.14, P=0.71, Int.- $F_{4,37}$=2.0, P=0.11, Stim.XInt.- $F_{4,37}$=0.2, P=0.94; **E**: Stim.-$F_{1,65}$=0.2, P=0.68, Int.- $F_{4,31}$=4.2, P=0.008, Stim.XInt.- $F_{4,32}$=0.1, P=0.94). (**F-G**) Mean±SEM tonic modulation in firing (last 400ms at high and low stimulus phases) normalised to peak phasic response for non-MR (**F**; n=94; combined data from 71 non-opponent, 13 L-ON and 10 S-ON) and MR units (**G**; n=30; combined data from 26 non-opponent, 1 L-ON and 3 S-ON). Data analysed by mixed-effects linear model with Sidak's post-tests (**F**: Stim.-$F_{1,688}$=0.0, P=0.96, Int.- $F_{4,397}$=9.0, P<0.001, Stim.XInt.- $F_{4,399}$=1.8, P=0.13; **G**: Stim.-$F_{1,202}$=0.4, P=0.54, Int.- $F_{4,123}$=19.7, P<0.001, Stim.XInt.- $F_{4,123}$=3.6, P=0.009). ** indicates P<0.01.

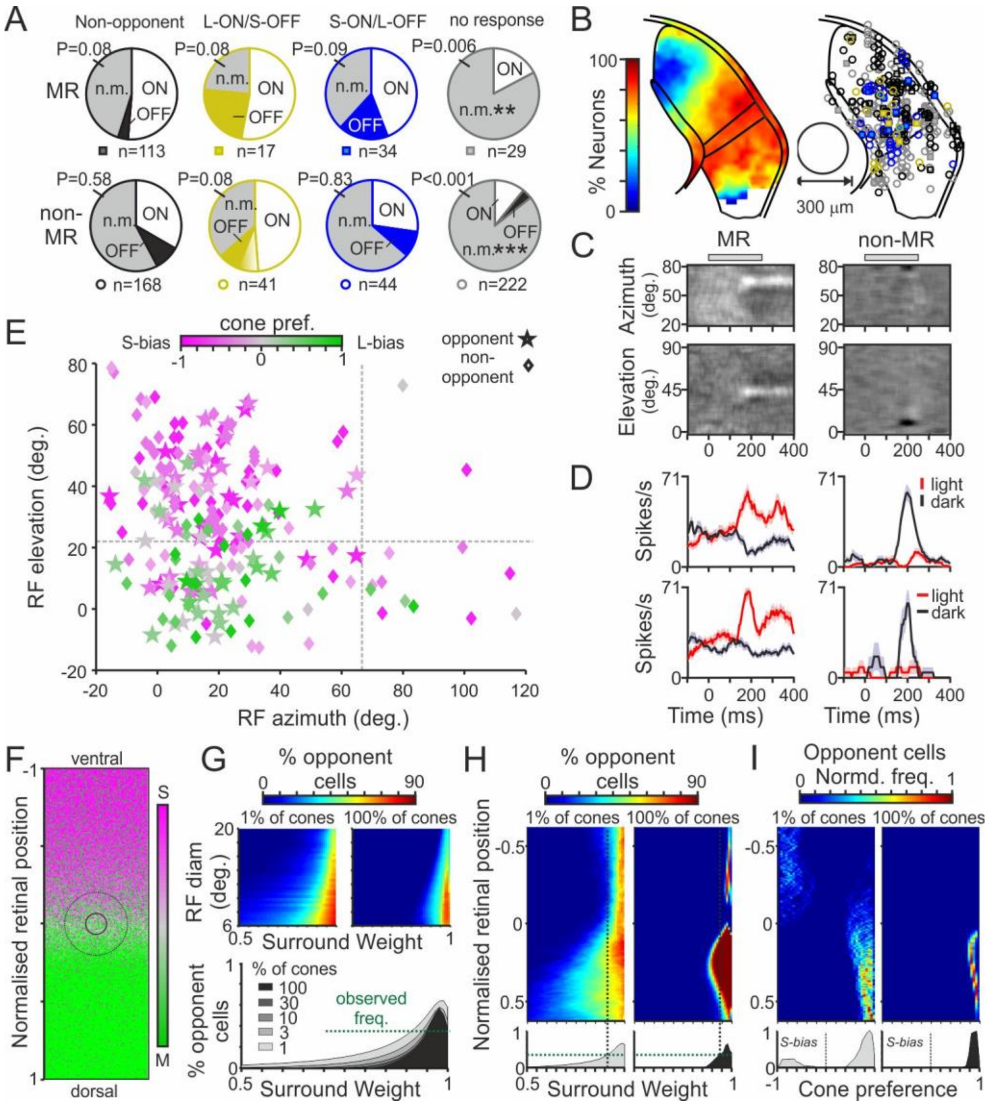

Figure S3. Receptive field positions and cone based responses. Related to Figure 3. (**A**) Distribution of RF properties (ON, OFF, ON/OFF or not mappable; n.m.) for MR and non-MR cells, subdivided based on responses to full field cone-isolating stimuli. Proportions of cells lacking mappable RFs were compared against that expected based on the proportion of visual field tested by Fishers exact test. (**B**) Left panels: proportions of neurons (n=92 MR and n=330 non-MR neurons) that lacked a mappable RF as a function of anatomical location (150µm radius binning), right panels: locations of individual cells. (**C**) Representative responses from MR (left) and non-MR (right) cells that lacked responses to full-field cone-isolating stimuli, conventions as in Figure 3A,B. (**D**) Mean±SEM firing rate following appearance of light and dark bars within the estimated RF centre for cells in **C**, conventions as in Figure 3C,D. (**E**) RF centre positions (defined relative to snout axis) for units in Figure 3E and F, colour coded according to cone preference from responses to full field cone-isolating stimuli (Figure 1). Dotted lines indicate projected midpoints of dorsal-ventral and nasal-temporal retinal axes. (**F-I**) Results from 'random-wiring' models of colour opponency (see Methods). (**F**) Example of modelled S- and M-cone opsin expression across a strip of retina and superimposed centre-surround RF profile (12°diameter, 36°surround). (**G**) Top panels show estimated percentages of opponent neurons as a function of RF diameter and surround weights from models where cells sampled from 1% (left) or 100% (right) of the cones within their RF. Lower panel shows the % of opponent neurons (averaged across all tested RF sizes) as a function of surround weight for cells that sampled randomly from varying proportions of cones. Dotted line shows the proportion of cells responding to full field stimuli with opponent responses from our data in Figure 1. (**H**) Estimated percentage of opponent neurons as a function of retinal position

and surround weight for 12°diameter RF cells (typical size for LGN neurons) sampling from 1% (left) or 100% (right) of cones within their RFs. Lower panels show average across the dorsal-ventral retina, with dotted lines indicating the surround weight producing a proportion of opponent cells that matches the experimental data. (**I**) Normalised distribution of cone preference as a function of retinal position for models that best matched observed proportion of opponent vs. non-opponent neurons in Figure 1 (12°diameter RF cells with surround weight = 0.91 and 0.94 in left and right panels; indicated by dotted lines in H).

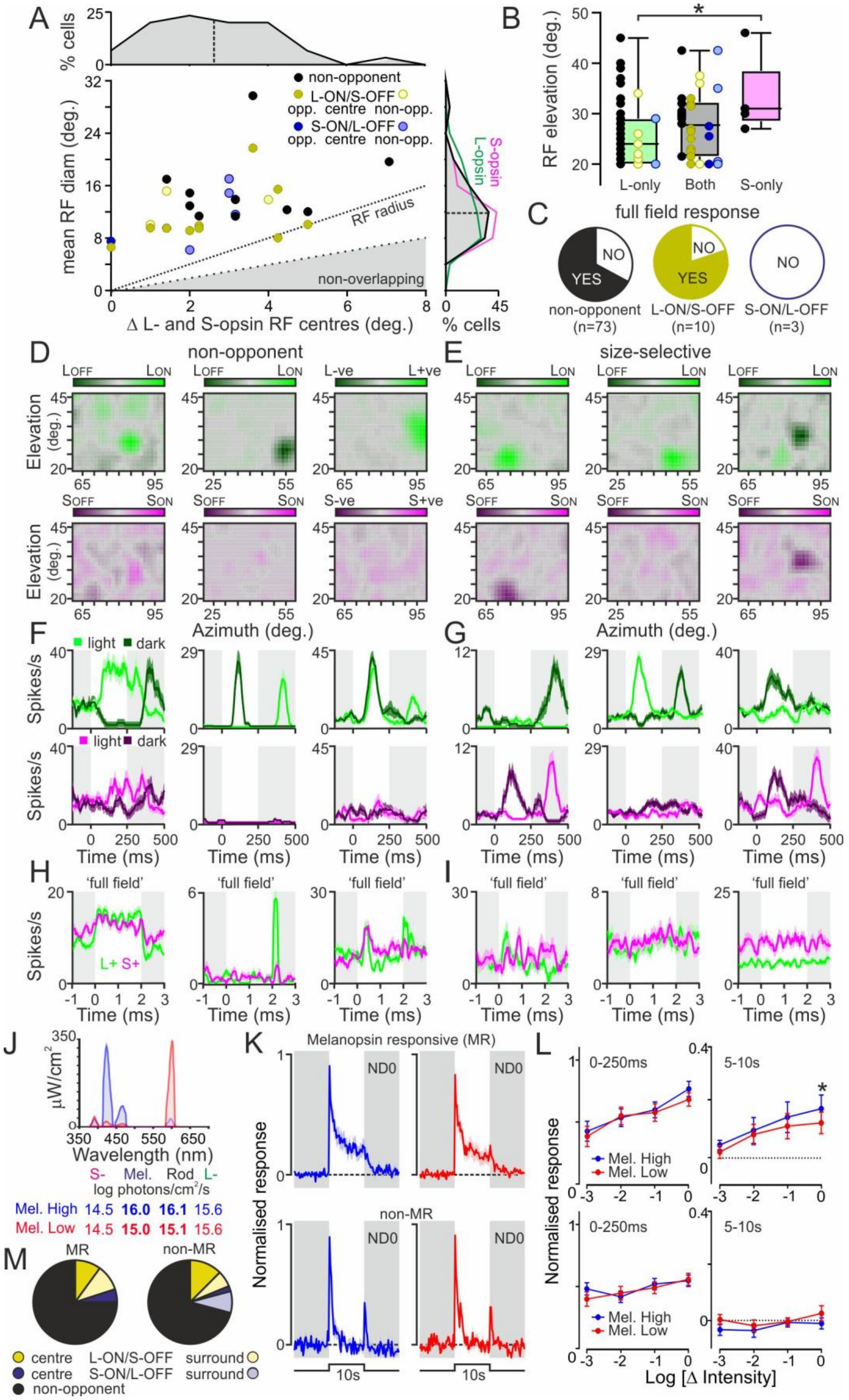

Figure S4. Properties of cone-dependent receptive fields in LGN neurons. Related to Figure 4. (**A**) Relationship between RF size and difference in apparent centre of L- and S-cone opsin RFs (for n=30 cells with mappable RFs for both). (**B**) Distribution of RF positions on elevation axis for neurons with mappable RFs to L- and/or S-opsin directed flashing squares. Data analysed by one-way ANOVA with Dunnett's post-tests ($F_{2,99}$=4.8, P=0.01). (**C**) Proportions of neurons with mappable cone-specific RFs that also responded to

full-field cone-specific stimulation. (**D**-**E**) L- and S-cone opsin dependent RF profiles for representative non-opponent neurons that did (**D**) or did not (**E**) exhibit robust responses to full field stimuli. (**F**-**G**) Mean±SEM responses to light and dark, L- and S-opsin directed squares (upper and lower panels respectively) appearing in the RF centre for neurons in **D**-**E**. (**H**-**I**) Mean±SEM responses to full-field 0.25Hz L- and S-cone opsin directed modulations for neurons in **D**-**G**. (**J**) Spectral power distribution of Mel. High and Mel. Low stimuli used to classify neurons as MR or non-MR. (**K**) Mean±SEM normalised firing rate profiles of MR (upper; n=20) and non-MR (lower, n=48) units presented with Mel. High and Mel. Low stimuli at maximum intensity. (**L**) Mean±SEM normalised responses of MR and non-MR units (as in **K**) during initial (left; first 250ms) and late (right; last 5s) of the 10s light steps. Data analysed by two-way RM ANOVA with Sidak's post-tests ($\mathbf{L}_{TopLeft}$: Stim.-$F_{1,19}$=1.2, P=0.28, Int.- $F_{3,57}$=5.1, P=0.004, Stim.XInt.- $F_{3,57}$=0.6, P=0.61; $\mathbf{L}_{TopRight}$: Stim.-$F_{1,19}$=8.7, P=0.008, Int.- $F_{3,57}$=2.7, P=0.05, Stim.XInt.- $F_{3,57}$=0.7, P=0.55; $\mathbf{L}_{BottomLeft}$: Stim.-$F_{1,47}$=0.4, P=0.53, Int.-$F_{3,141}$=2.5, P=0.06, Stim.XInt.- $F_{3,141}$=1.0, P=0.39; $\mathbf{L}_{BottomRight}$: Stim.-$F_{1,47}$=0.9, P=0.47, Int.-$F_{3,141}$=7.0, P=0.01, Stim.XInt.- $F_{3,141}$=1.5,P=0.21). * indicates P<0.05. (**M**) Classification of RF type for identified MR and non-MR neurons.
